# A review of *Sarcoptes scabiei*: past, present and future

**DOI:** 10.1186/s13071-017-2234-1

**Published:** 2017-06-20

**Authors:** Larry G. Arlian, Marjorie S. Morgan

**Affiliations:** Department of Biological Sciences, Wright State University, 3640 Colonel Glenn Hwy, Dayton, OH 45435 USA

**Keywords:** *Sarcoptes scabiei*, Biology, Host-seeking behavior, Infectivity, Nutrition, Host-parasite interaction, Immune modulation, Diagnostic test, Vaccine

## Abstract

The disease scabies is one of the earliest diseases of humans for which the cause was known. It is caused by the mite, *Sarcoptes scabiei*, that burrows in the epidermis of the skin of humans and many other mammals. This mite was previously known as *Acarus scabiei* DeGeer, 1778 before the genus *Sarcoptes* was established (Latreille 1802) and it became *S. scabiei*. Research during the last 40 years has tremendously increased insight into the mite’s biology, parasite-host interactions, and the mechanisms it uses to evade the host’s defenses. This review highlights some of the major advancements of our knowledge of the mite’s biology, genome, proteome, and immunomodulating abilities all of which provide a basis for control of the disease. Advances toward the development of a diagnostic blood test to detect a scabies infection and a vaccine to protect susceptible populations from becoming infected, or at least limiting the transmission of the disease, are also presented.

## Background

The ancestral origin of the scabies mite, *Sarcoptes scabiei*, that parasitizes humans and many families of mammals is not known. Likewise, how long ago the coevolution of *S. scabiei* with specific host mammals began and how this has evolved over time is unknown. However, acarologists and mammalogists using molecular tools and genomic information may clarify these questions in time.

Roncalli [1] and Friedman [2] provide a history of scabies in humans and in veterinary medicine from biblical times to the early 1900s. The earliest written reference to a skin disease of humans and other mammals that could be scabies appears in Leviticus in the Bible (1200 BCE) [1]. According to Friedman [2], the causal relationship between the itch mite *Acarus scabiei* (now *Sarcoptes scabiei*) and the disease in humans was discovered by Bonomo and Cestoni in 1687 and “it marked the establishment for the first time in the history of medicine of a definitely known cause for any of the diseases of man”. Today, scabies is a neglected, globally prevalent, contagious skin disease of humans and many domestic and wild mammals and causes significant morbidity and mortality.

### Classification of scabies mites

*Sarcoptes scabiei* was initially placed in the genus *Acarus* and named *Acarus scabiei* DeGeer, 1778. As mite nomenclature has evolved, so has the classification of *S. scabiei*. *Sarcoptes scabiei* is now placed in the superfamily Sarcoptoidea and family Sarcoptidae along with many other ectoparasitic mites of mammals. Among the acari, *S. scabiei* belong to the superorder Acariformes, order Sarcoptiformes, suborder Oribatida, Infraorder Desmonomata and the group (hypoorder) Astigmata (along with the house dust mites *Dermatophagoides farinae*, *D. pteronyssinus* and *Euroglyphus maynei*) [3].

**Fig. 1:**
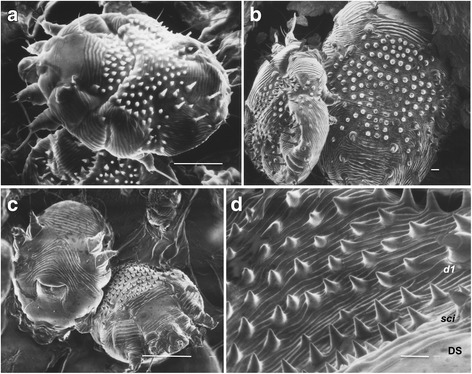
Scanning electron micrographs of female *Sarcoptes scabiei* var. *hominis* (**a**), *S. scabiei* var. *suis* (**b**) and *S. scabiei* var. *canis* (**c**, **d**) showing dorsal spines, coarse cuticular striations and internal scapular lamellate setae (*sci*), dorsal setae (*d1*) and the dorsal shield (DS). *Scale-bars*: **a**, 100 μm; **b**, 10 μm; **c**, 100 μm; **d**, 10 μm

The family Sarcoptidae contains three subfamilies (Sarcoptinae, Teinocoptinae, and Diabolicoptinae) including 16 genera and 118 species, that are all inhabitants of the skin of mammals [4, 5]. The subfamily Sarcoptinae includes the four genera *Sarcoptes* (1 species), *Prosarcoptes* (3 species), *Trixacarus* (3 species) and *Kutzerocoptes* (1 species). Both *Sarcoptes* and *Trixacarus caviae* look much alike and may be confused. *Trixacarus caviae* is a parasite of guinea pigs and is much smaller than *Sarcoptes* [6]. *Trixacarus caviae* can cause pruritic dermatitis in humans that hold or handle infested guinea pigs [7]. In addition to their size differences, a few other features easily distinguish *Sarcoptes* from *T. caviae*. The dorsal setae of *T. caviae* females are simple while those of *S. scabiei* are cone- and spine-shaped and the dorsal scales of *T. caviae* are more extensive than *S. scabiei* and extend to the posterior of the idiosoma [7]. The dorsal setae *sci, l1*, and *d1* of *T. caviae* are not lamellate as they are in *S. scabiei* (Fig. 1).

### Morphology

Detailed descriptions of *S. scabiei* with schematic diagrams have previously been published [2, 4–6]. Briefly, *S. scabiei* has an oval tortoise-like body (idiosoma) that is ventrally flat and dorsally convex (Fig. 1). The dorsal idiosoma bears stout lateral (*l*) and dorsal (*d*) setae, cuticular spines and coarse, transversely ridged, cuticular striations. The dorsal setae *sci, l1* and *d1* are lamellate (Fig. 1d).

**Fig. 2:**
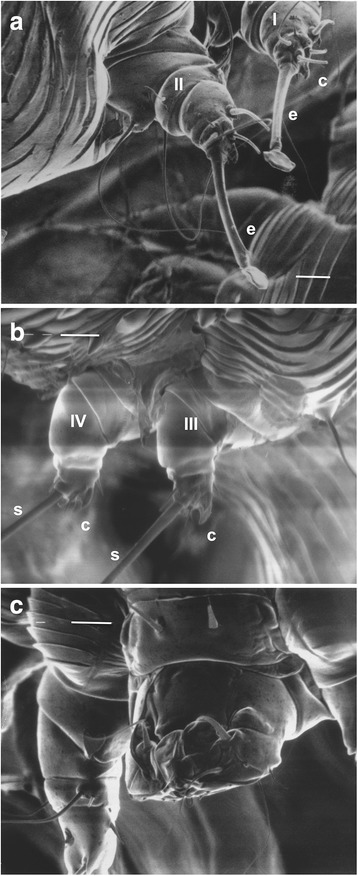
Scanning electron micrographs of female *Sarcoptes scabiei* var. *canis*. **a** Legs I and II showing tarsus with claws (*c*) and stalked empodium (*e*) that terminates in a pad. **b** Legs III and IV showing two claws (*c*) and long seta (*s*) on the tarsus. **c** Gnathasoma (pedipalps and chelicerae) and leg I. *Scale-bars*: **a**, 10 μm; **b**, 10 μm; **c**, 10 μm

All legs of both females and males are short and stubby (Figs. 1, 2). Legs III and IV of both sexes do not extend beyond the lateral-posterior margin of the idiosoma while legs I and II extend beyond the anterior margin of the idiosoma with the tarsus that bears a stalked empodium that terminates in a pad (Fig. 2a). Legs IV of males also bear a stalked empodium that terminates in a pad. All other legs of males and females (legs III and IV of females and legs III of males) terminate in long setae. All terminal segments of the legs of both males and females have claws (Fig. 2). Two spur-like claws are present on the terminal segments of legs I, II, III and IV of females. Males have two spur-like claws on legs I, II and III and one on leg IV.

**Fig. 3:**
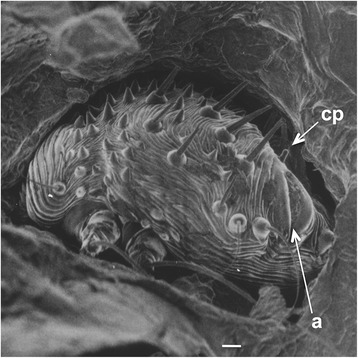
Scanning electron micrograph (posterior view) of female *Sarcoptes scabiei* var. *canis* in a burrow in the stratum corneum showing the dorsal terminal anal opening (*a*) and copulatory papilla (*cp*) of the bursa copulatrix. *Scale-bar*: 10 μm

The gnathosoma (capitalum) consists of short, stout chelicerae and pedipalps (Fig. 2c). The anal opening of females is posterior/dorsal with the nipple-like papilla of the bursa copulatrix situated anterior to the anal opening (Fig. 3). Average fresh and dry weights of females are 5.62 ± 1.25 μg and 2.8 ± 0.86 μg, respectively [8]. Males are much smaller with wet weight 1.49 ± 0.59 μg and dry weight 0.39 ± 0.16 μg [8].

### Animal models to study scabies mite biology

The lack of large numbers of *S. scabiei* var. *hominis* mites from humans has limited extensive studies on the biology of human scabies mites. Occasionally, large numbers of mites can be obtained from a patient with crusted scabies (Norwegian scabies) for this purpose. Thus, many biological, host interaction, immunological, proteomic and genomic studies must rely on animal strains of scabies mites and a host animal model such as rabbits or pigs. Where direct comparisons have been possible, var. *canis* and var. *hominis* mites appear to have similar biology.

Two animal models for the propagation of scabies mites are now available for studying both the mite, its immune-modulating ability, and the host-parasite interactions and are responsible for much of the knowledge of scabies mites gained over the last 30 years. A rabbit/canine scabies model was developed many years ago [9]. However, it is not clear that this is a trans-species model. It may well be a rabbit/rabbit model. The original source of the scabies mites used to experimentally infect the rabbits was roaming dogs. But these dogs may have been infected from rabbits that the dogs preyed upon for food (prey-to-predator transfer is discussed under “Cross-host species transfer”). The ease by which the rabbits were infected with mites collected from roaming dogs suggests this possibility but there is no way of knowing the origin of the mites. The other model used in more recent studies utilizes pig mites (var. *suis*) and a pig host [10].

### Life-cycle

The developmental stages of *S. scabiei* consist of egg, larva, protonymph, tritonymph and adult. This life-cycle is typical of that for other astigmatid mites. There is considerable difference in the reported durations of the life-cycle of *S. scabiei* var. *hominis*. Reported durations of the life-cycle for scabies mites from humans are 12 to 17 days [11], 17 to 21 days [12], 7 to 10 days [13], 9 to 15 days [14] and about 15 days [2]. In the historic published literature, it is suggested that females produce 40–50 or more eggs over a life span of 26–40 days [2].

A systematic in vivo study of the life-cycle of *S. scabiei* var. *canis* using a rabbit model reported the duration from egg to adult to be 10 to 13 days [15]. Larvae emerged from eggs after 50 to 53 h of incubation. Duration of the larval stage was about 3–4 days while for the protonymphal and tritonymphal stages it was about 2–3 days for each life stage. Comparable times of 10–15 days for the life-cycle are reported for var. *suis* in pigs [16].

The reasons for the variations in the reported durations of the life-cycle of *S. scabiei* are unknown. The variations are likely attributed to the difficulty of observing their development in vivo in the skin. Different observation methods used to obtain this information, different temperature and relative humidity conditions during the observation periods, and observation of scabies mites from different hosts may be contributing factors.

### Host seeking

*Sarcoptes scabiei* mites seek the source of stimuli originating from the host when they are off the host but in close proximity to it. This behavior may facilitate their finding a host if they are dislodged from it and contaminate the host environment. Thus, direct contact with an infested host may not be required for humans and other mammals to become infected with *S. scabiei*. In the case of human scabies, live mites in bedding, furniture, toys, and clothing can be a source of infection. *Sarcoptes scabiei* var. *hominis* have been recovered from laundry bins in a nursing home [17]. For wild and domestic mammals, shared or common bedding places, stalls in barns and corrals could serve as a source of infecting scabies mites.

Experiments conducted using var. *canis* mites, showed that female mites placed on a metal wire (1 mm in diameter, positioned perpendicular to and touching the host) at varying distances from the host walked along the wire toward the host [18]. More than 68% of female mites tested moved towards the host when placed 4.9 cm (1.93 in.) away from it while 100% moved in this direction when placed 4.2 cm (1.65 in.) away. About 20% of the test mites migrated to the host when placed 11.2 cm (4.41 in.) away. Thus, the ability to perceive and respond to a host diminishes with increasing distance from the source. In these experiments, the host stimulus that induced the response could have been body odor and heat emanating from the host and/or CO_2_ in exhaled breath.

Additional experiments showed that mites would seek a thermal stimulus source without a host present [18]. More than 83% of the female mites sought the heat source that was 5.6 cm (2.2 in) away. However, females responded equally (50% to each) to both an artificial heat stimulus and odor from host skin when given a choice of the two simultaneously and in close proximity (2.5 cm = 1 in) to the stimuli. At a distance of 6.5 cm (4.41 in) from both stimuli, 38% of mites chose stimuli from a live host, 5% chose the artificial heat stimulus at 32 °C and 57% chose neither which suggested that the host odor was a stronger attractant than heat when the two stimuli were offered together. The 57% that did not respond to either may have been confused by the two stimuli they responded to when each was offered separately but now offered together but from opposite directions. In other two-choice experiments, scabies mites chose air containing host odor in the absence of CO_2_ so CO_2_ is not required to induce a response.

Mellanby et al. [19] observed that *S. scabiei* var. *hominis* placed in a temperature gradient between 20 °C and 30 °C exhibited a similar thermotactic response. *Sarcoptes scabiei* var. *hominis* placed below 24 °C moved towards the hotter part of the gradient. No mites moved to areas below 24 °C.

These experiments clearly show that scabies mites in the environment near the host perceive stimuli (odor, body temperature) from the host and will seek the source.

### Photoresponse

The ability to detect light and its intensity may be important factors along with host odor and warm body temperature in host detection by *Sarcoptes* mites. Photoresponse by scabies mites has not been extensively investigated. *Sarcoptes scabiei* var. *hominis* was previously reported to be unresponsive (positive or negative) to a light stimulus although no data were presented [19]. In contrast, *S. scabiei* var. *canis* is attracted to fluorescent overhead room light at 63 fc (678 lm/m^2^) and 100 fc (1076 lm/m^2^) (Table 1). The majority of mites that had emerged from a scabietic skin crust migrated to the light rather than remain in the dark of an observation arena.

**Table 1:** Distribution of *Sarcoptes scabiei* var. *canis* in a light/dark choice arena following release in the dark

Light intensity (foot candles)	*n*	Exposure time (h)	Replicates	% Mites in light	% Mites in dark
63	488	1	2	53	47
	768	3	2	62	38
	455	5.5	1	77	23
100	629	1	5	21	79
	472	3	5	39	61
	394	15.5–16.0	5	63	37

The lighted side of the choice arena was uniformly lighted with fluorescent room light at 63 or 100 fc (678 or 1076 lm/m^2^) and the distribution of live mites was determined after specific exposure times

### Survival off the host

Contact with an infected host is generally considered to be the primary means by which an individual becomes infected with scabies. This idea is based largely on studies by Mellanby [11] who found that only 4 individuals of 272 tested became infected with scabies after sleeping in beds used by heavily infected patients. However, the role of fomites, the survival of mites off the host, and their infectivity in the transmission of scabies has never been extensively investigated. The ability of scabies mites to survive and to remain infective while off the host are key factors in the infection of hosts from mites in the environment.

**Fig. 4:**
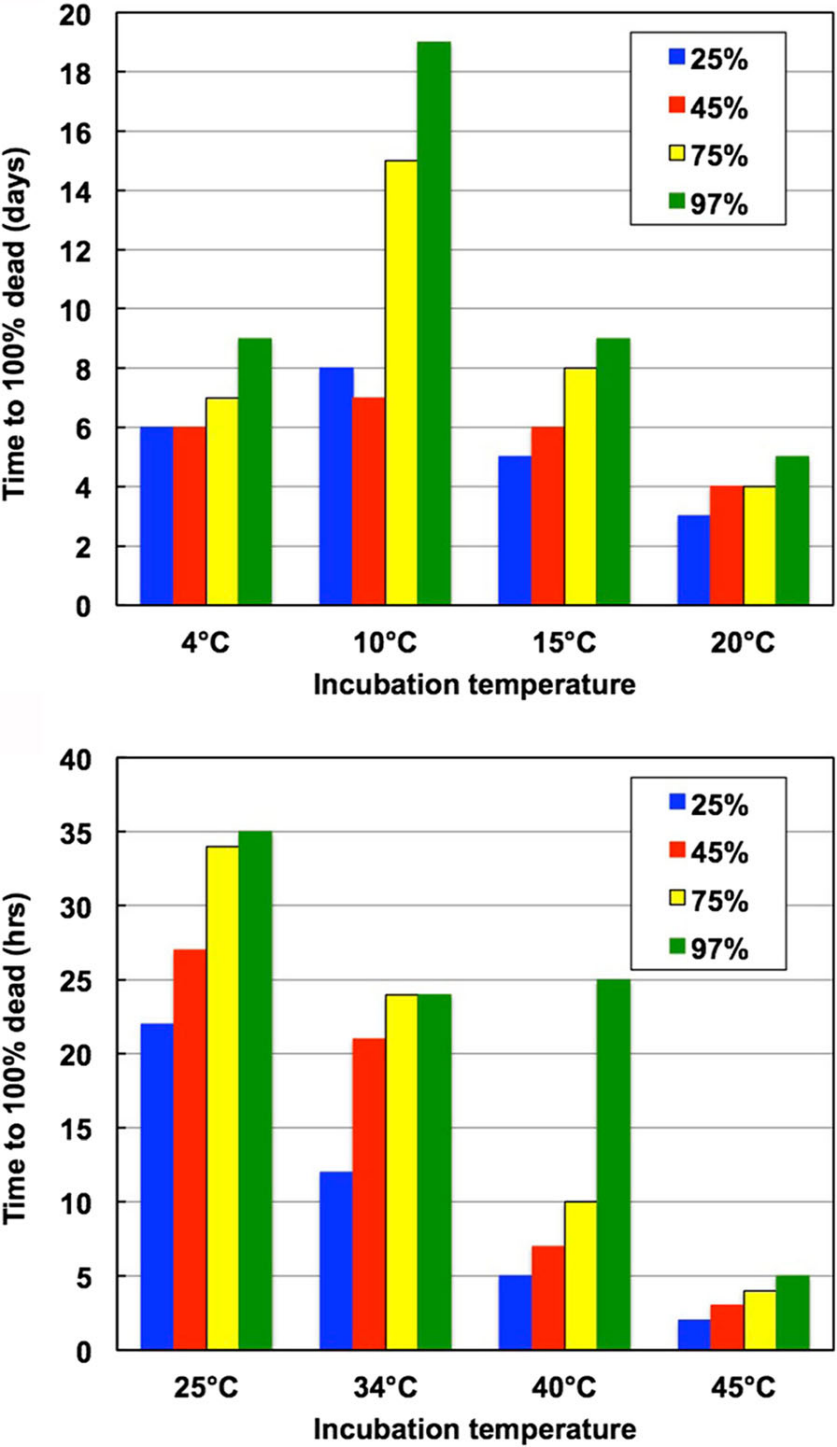
Observed time to achieve 100% mortality in test populations of female *S. scabiei* var. *canis* mites exposed to specific combinations of temperature and relative humidity (RH). The number of mites in each test group ranged from 8 to 26. Data from [20]

Animal strains of *S. scabiei* are suitable models to determine survival and retained infectivity for mites in the host environment. A study by Arlian et al. [20] found that *S. scabiei* var. *canis* females survived for a week or more when held at 15 °C (59 °F) and relative humidity (RH) above 75% (Fig. 4). At a warmer temperature of 25 °C (77 °F), females survived 1–2 days at all of the RHs tested (Fig. 4). Male survival time off the host was much shorter compared to females. These studies showed that generally, warmer temperatures drastically reduced survival time at each humidity. In this study, the mites clearly died of dehydration due to their inability to maintain their water balance (an issue addressed later). Higher RH and temperatures below 20 °C allowed for longer survival times.

On the other hand, *S. scabiei* var. *hominis* fasting female mites can survive 19 days at 10 °C and 97% RH and 8 days at 10 °C and 25% RH [20]. *Sarcoptes scabiei* var. *hominis* that had been held off the host at alternating 12 h periods of 4 °C or 10 °C and 95% RH and room conditions (21 °C and 45% RH) for 4 days remained infective and would still penetrate the skin of a rabbit [20].

Freezing might be an option to kill scabies mites in items such as stuffed and hard toys, small pillows, and bedding. Freezing female var. *canis* mites at -25 °C and 50% RH for 1.5 h resulted in 100% mortality. After 1 h of freezing, 23% of test mites survived but they did not initiate penetration when placed back on the host skin [20]. It is not known how long these mites can survive at -15 °C to -17 °C, the temperature of a typical home freezer.

Mellanby et al. [19] determined the thermal death point of *S. scabiei* var. *hominis* females exposed to various temperatures for 10 and 30 min and 0–90% RH. The lethal temperatures were 49 °C (120 °F) in 10 min and 47.5 °C (117.5 °F) in 30 min. After extraction from the host, they found survival to be 84.7, 30.5, 6.8, 1.7 and 0% for 1, 2, 3, 4 and 5 days of exposure, respectively, to 21.0–25.5 °C and 90% RH. Survival at 24–25 °C and 30% RH was 63.5, 6.8 and 0% after 1, 2, and 3 days of exposure, respectively. However, 61, 52, 35, 30 and 26% of mites survived 5, 6, 7, 9 and 11 days, respectively, at 90% RH but few mites survived 14 days. In contrast, only 4 and 2% of mites survived 2 and 4 days, respectively at 30% RH. Thus, high RH and low temperature also prolong var. *hominis* mite survival off the host. The mites did exhibit some ability to survive freezing temperature. All test mites survived 2 days at 0 °C and 28.6% survived 8 days.

### Infectivity

An important aspect of environmental (fomite) transmission is how long mites remain infective when off of the host. As previously mentioned, survival time off the host is directly related to ambient relative humidity and temperature. Studies using a rabbit host and *S. scabiei* var. *canis* strain found that most mites held off the host for 24 h would initiate penetration of the skin very soon after being placed back on the skin of a host [20]. They became half to completely submerged in a newly formed burrow within an hour. Female var. *canis* mites begin penetration in about 20 min while males, nymphs and larvae initiate burrowing in less than 5 min after being placed on the skin. Eighty percent of females held off the host for 36 h at 75% RH and 22 °C could completely penetrate the host skin within an average time of 97 min. As noted earlier, females held off the host for 24 h penetrated the skin in much less time while no mites survived 48 h at 75% RH and 22 °C. For comparison, live human scabies mites (var. *hominis*) obtained from bed linen slept in by patients heavily infected with scabies could initiate penetration of the skin within 10 min after placement on a rabbit and be fully penetrated within 31 min [20].

Thus, in homes, schools and nursing homes, extensive cleaning, disinfection and laundering should not be required to eliminate scabies mites in dry climates. Leaving a bed, bedroom, bedding and clothing isolated for 48 h at room temperature should result in the death of scabies mites. In dry climates (< 50% RH) mites likely survive and remain infective for less than 36 h.

### Penetrating the host skin

When scabies mites infect a host, they first must penetrate the stratum corneum of the epidermis of the skin. It is a tedious task to observe this process microscopically. However, all life stages of both *S. scabiei* var. *canis* and *S. scabiei* var. *hominis* have been experimentally observed under the microscope while they penetrated the host skin [20]. Mites placed on the skin secrete a clear fluid (presumably saliva) that forms a pool around their body. It appears that the stratum corneum is dissolved (lysed) and the mite sinks into a depression in the skin. As it sinks, legs I and II seem to move in a tortoise like fashion (digging, crawling/swimming motion). The action of the legs propels the mite forward as a tunnel-burrow in the stratum corneum is formed. The time to become completely submerged in the stratum corneum is amazingly short as detailed above.

### Water balance

Fresh bodies of female and male *S. scabiei* var. *canis* are 57 ± 12% and 69 ± 18% water by weight, respectively [8]. The procurement of host intercellular fluid appears to be necessary for mites to obtain sufficient water to maintain their water balance [8]. Some mite and tick species have the ability to maintain water balance without ingestion of water by absorbing water from unsaturated ambient air when the RH is above a critical level [21]. For example, the related house dust mites, *D. farinae* and *D. pteronyssinus*, have an active mechanism for extracting water from unsaturated air that is above about 70% RH to replenish all water that is lost by evaporation, secretions and other body processes (excretion and defecation) [22, 23]. Dehydrated house dust mites and ticks gain water and thus body weight when placed in humid air at humidity above the critical humidity level. Scabies mites appear to be incapable of active uptake of enough water from unsaturated air in their surroundings on the skin surface or in a burrow to maintain water balance and when dehydrated cannot recover the lost water from nearly saturated air (Table 2). Scabies mites lose body water and dehydrate when held off the host at 97.5% RH [8]. Thus, unlike the related house dust mites, scabies mites are unable to absorb water vapor from unsaturated ambient air to satisfy their water requirements. Females lose 30–34% of their body water mass when held for 16 h at 97.5% RH. Thus, they must ingest/imbibe from the host the water needed to maintain their water balance.

**Table 2:** Weights of female *Sarcoptes scabiei* var. *canis* immediately after removal from the host (fresh weight) and their weights after being held off the host at 97.5% RH for 16 h for three groups of mites

Group	Fresh weight (μg/mite)	Weigh after 16 h at 97.5% RH (μg/mite)
Group 1 (*n* = 30)	5.74 ± 1.84	4.02 ± 1.18
Group 2 (*n* = 8)	6.32 ± 1.48	4.15 ± 1.29
Group 3 (*n* = 13)	6.54 ± 1.28	4.47 ± 0.95

Data from [8]

The metabolic rate (oxygen requirements) of scabies mites has been determined using the Cartesian diver technique and is insufficient to provide a scabies mite with significant amounts of metabolic water [8].

### Nutrition procurement

Scabies mites reside in burrows that they make in the nonliving stratum corneum of the epidermis of mammalian skin. It was once believed that these mites feed on lysed stratum corneum. However, subsequent studies suggested that the mites ingest intercellular fluid (lymph) that seeps into the burrow around the mite mouthparts as they burrow deep in the stratum corneum near the living tissue (cells) of the lower epidermis. Several lines of evidence support this. Both scanning electron microscopy and light microscopy show that mites in the skin stratum corneum reside at the interface of the stratum lucidum and stratum granulosum [24–26] where intercellular fluid is close to the mite location and can seep into the burrow. Mites appear to burrow downward towards the dermis to maintain this location as the basal layer of cells proliferates and the upper layer of the dry stratum corneum is pushed toward the skin surface. Demonstrating the presence of host IgG antibody in the esophagus and midgut of fresh scabies mites removed from the host is evidence that these mites ingest host serum [27]. They appear to be unable to digest IgG and possess a relatively limited repertoire of enzymatic activities which is consistent with a serum diet [28].

Scabies mites are aerobic. Females and males utilize 0.00206 μl O_2_/h/mite and 0.00076 μl O_2_/h/mite, respectively [8]. Extrapolating from this and using a rabbit model host with 30% of the body surface area infected, the mites’ energy demands on the host was determined to be insignificant even though these mites obtain all of their nutrition from the host.

### Location of scabies mites on the host body

Human scabies mite infections are typically localized in specific parts of the body. Hands, wrists and elbows are the most commonly infected sites in adult patients [11]. However, genitals, feet, buttocks, axillae, breasts, and waistline are also favored sites of infection [11].

It is not known why certain areas of the body are more commonly infected than others. Hand and wrist infections may simply be the result of touching infected persons and handling mite-contaminated materials. However, the distribution patterns suggest that the mites select favored areas and these areas may be preferred in part because of the lipid composition and other site-specific factors of the skin in these areas.

Arlian & Vyszenski-Moher [29] evaluated the response of all life stages of *S. scabiei* var. *canis* to multiple concentrations of 21 lipid compounds that are typically found in or on the epidermis of human skin. They found that a variety of these lipids attracted scabies mites including 13 fatty acids, 5 fatty acid methyl esters, cholesterol, squalene and tripalmitin. The molar concentration that elicited the greatest response varied depending on the compound. A comparison of the different life stages showed that the life stage response depended on the compound and the molar concentration of that particular compound. In some cases, one life stage responded to a concentration of a compound while another did not. Overall, females were the most responsive.

Lipid contents and lipid mixtures of the skin vary in different anatomical areas of the body. Therefore, it is difficult to understand the significance of the scabies mite response to different molar concentrations of these isolated lipid compounds. The predilection of mites for particular areas of the body is not understood but probably involves the interaction of multiple factors in and on the skin. The study clearly demonstrated that scabies mites were attracted to many of the skin lipids that were tested and therefore lipids may be involved in the attraction of the mites to favored areas of the body.

### Aggregation, sex and assembly pheromones

All life stages of scabies mites have been observed to leave their burrows and wander around on the skin. Even with low numbers of mites present on a host, males and females find each other and mate. Pheromones emitted from the mites are probably involved in these processes. Guanine, other purine compounds and other nitrogenous wastes of arachnids and phenolic compounds have been shown to be assembly-attraction-attachment pheromones or sex pheromones in ticks and mites [30–34]. Other acari such as *S. scabiei* probably produce and respond to similar compounds. In a study by Arlian & Vyszenski-Moher [35], 10 nitrogenous metabolites and 3 phenolic compounds were offered separately to all life stages of *S. scabiei* var. *canis.* Guanine, purine, adenine, allantoin, hypoxanthine, xanthine, uric acid, ammonium chloride, ammonium nitrate and ammonium sulfate all attracted a significant number of scabies mites. All life stages were also significantly attracted to the three phenolic compounds that were tested. These included 2,6-dichlorophenol, methyl salicylate and 2-nitrophenol. Females responded to the most concentrations of the various compounds while males responded to the least. It appears that nitrogenous and phenolic compounds may act as pheromones for scabies mites as they do for other acari.

### Host-parasite interactions (immune modulation)

As scabies mites burrow into the skin, they release substances that induce inflammatory and immune responses by the host as well as substances that can depress certain aspects of these responses allowing the mites to circumvent the host protective mechanisms. The latter helps the mites initially survive in the host skin and establish a population. There is a complex interaction at the mite-skin interface that determines the balance between the two competing processes over time and thus the course of an infection and the eventual manifestation of clinical symptoms. Because skin symptoms are not experienced for four or more weeks in a primary infection, it appears that early in an initial infection the innate/immune events are depressed but over time, as the mites proliferate and a population is established, there is a shift to more dominant inflammatory and immune responses and symptoms of the disease are manifested. What is responsible for the shift is not known, but the evidence is overwhelming that scabies mites, like so many other parasites, can modulate various aspects of the mammalian innate and adaptive immune responses. The mites’ immune modulating abilities that have been identified include anti-inflammatory, anti-immune and anti-complement activities.

Because of the mite’s location at the intercellular fluid interface in the epidermis, soluble substances (including saliva, molting enzymes and hormones, feces and nitrogenous excretory materials) from the mites containing antigenic and pharmacological activities diffuse into the fluid bathing the cells of the epidermis and dermis. These substances induce responses from the cells in these tissues including keratinocytes, fibroblasts, macrophages, mast cells, lymphocytes, Langerhans cells and other dendritic cells, and endothelial cells of the microvasculature.

In vitro studies show that molecules in a whole body extract of *S. scabiei* var. *canis* stimulated cultured normal human epidermal keratinocytes to greatly increase secretion of interleukin-6 (IL-6) and vascular endothelial growth factor (VEGF), and to slightly increase secretion of granulocyte colony stimulating factor (G-CSF) [36]. No effect on the secretion of IL-1α and IL-1β was observed. In addition, the scabies mite extract caused decreased secretion of IL-1 receptor agonist (IL-1ra) and IL-8 by these cells after they had been stimulated with lipopolysaccharide (LPS). Likewise, substances in this extract stimulated cultured normal human dermal fibroblasts to increase secretion of IL-6, IL-8, G-CSF and VEGF. In the skin, the collective, up-regulation of IL-6 and VEGF, accompanied by down-regulation of IL-1ra would promote inflammation (although IL-6 can have both pro-inflammatory and anti-inflammatory functions). VEGF promotes increased vascular permeability that would benefit the mite by providing more nutritional serum for it to ingest.

A follow-up study determined that *S. scabiei* var. *canis* extract in the presence of proinflammatory cytokines (IL-1α, IL-1β, and tumor necrosis factor-α [TNFα]) together with IL-17, that are likely present in the scabietic lesions in vivo*,* still downregulated IL-8 levels in the supernatants of both human epidermal keratinocytes and dermal fibroblasts at 8 h but this effect disappeared over time [37]. Granulocyte-macrophage colony stimulating factor (GM-CSF) secretion from fibroblasts was also diminished. This study also found that molecules in the scabies mite extract induced secretion of growth related oncogene-α (GROα), transforming growth factor-α (TGFα), and cutaneous T cell-attracting chemokine (CTACK) from keratinocytes and confirmed the enhanced secretion of IL-6 and G-CSF from fibroblasts. The shifting levels of IL-8 in the supernatants are likely due to the presence of two different molecules in the mite extract: one that stimulates IL-8 production and the other that binds to IL-8 as it is produced [37]. Ticks are known to produce an IL-8 binding protein (Evasin-3) that selectively binds and neutralizes IL-8 to inhibit the recruiting of neutrophils to the site of parasite invasion [38–40].

In the living skin, keratinocytes, fibroblasts and microvascular endothelial cells interact with one another within a collagen matrix and communicate using cytokines, chemokines and contact. These interactions could influence the response of individual cell types to scabies mite extract. Furthermore, living mites may release substances that are quantitatively and qualitatively different than components of a whole body extract. Thus, to simulate real skin, human skin equivalents (HSE) were challenged with *S. scabiei* var. *canis* extract and with living mites [41]. The HSE model consists of a dermis of fibroblasts in a collagen matrix located below an epidermis consisting of a layer of stratified keratinocytes. The HSE model allows for the interaction of fibroblasts and keratinocytes that occurs in vivo when stimulated by scabies mites and their products that is not possible to observe in cell monoculture experiments. Living mites burrowed into the stratum corneum of these skin equivalents just as they do in the intact skin of a host [41]. Burrowing mites induced the secretion of CTACK, thymic stromal lymphopoietin (TSLP), IL-1α, IL-1β, IL-1ra, IL-6, IL-8, monocyte chemoattractant protein-1 (MCP-1), GM-CSF, and macrophage colony stimulating factor (M-CSF) onto the surface of the HSE. The main difference between the response to mite extract of monocultured keratinocytes and fibroblasts was that the HSE produced significant amounts of the proinflammatory cytokines IL-1α and IL-1β and their inhibitor IL-1ra in response to burrowing mites. The upregulation of IL-1ra may be an important part of the mechanism used by these mites to depress the host’s inflammatory response allowing a mite population to become established.

Studies of gene expression in the HSEs also confirmed and expanded on the results of the in vitro monoculture experiments. Scabies mites burrowing into in the HSEs induced upregulated expression of 189 genes and downregulated expression of 152 genes in the keratinocytes and fibroblasts in these HSEs [42]. Genes for a number of cytokines were upregulated, paralleling the cytokine secretion profiles reported above [41]. Of particular note was the upregulation of IL-20. This cytokine promotes the proliferation of keratinocytes and its upregulation may contribute to the development of the scaly and crusted skin that is characteristic of chronic scabies. Additionally, the most upregulated gene was that for type 1 keratin, the predominant structural protein in keratinocytes. Many other genes were down-regulated when mites burrowed in the HSEs, including several members of the cytochrome p450 family. The reader is referred to [42] for the complete list of genes that were differentially expressed in response to burrowing *S. scabiei* mites in the HSE model.

Cultured human dermal microvascular endothelial cells of the skin stimulated with *S. scabiei* var. *canis* extract decreased the TNFα-induced expression of the cell adhesion molecules E-selectin and vascular cell adhesion molecule-1 (VCAM-1) and the levels of IL-8 while it increased the expression of the cytokine receptor CXCR-1 [43]. A subsequent study confirmed that scabies mite extract was still able to decrease TNFα-induced VCAM-1 expression in the presence of some proinflammatory mediators (e.g. histamine, leukotriene B4 and IL-1 cytokines) that may occur in scabietic lesions in vivo [44]. IL-1α and IL-1β induced IL-6 secretion was also decreased in the presence of scabies mite extract.

In vitro studies show that peripheral blood mononuclear cells (PBMCs, mostly monocytes) from healthy human donors stimulated with *S. scabiei* var. *canis* extract upregulate secretion of IL-1β, IL-6, IL-8, and TNFα [45]. Dendritic cells derived from these monocytes downregulated secretion of IL-6 and IL-8 after they had been stimulated with LPS to induce secretion of these cytokines [45]. Likewise, Walton et al. [46] found that PBMCs from patients with ordinary scabies produced higher levels of IL-5 and IL-13 and lower levels of interferon gamma (IFNγ) when stimulated with the recombinant human cysteine protease Yv5032C08. PBMCs from patients with ordinary or crusted scabies also showed a strong proliferative response when stimulated with this molecule.

In another study, mixed populations of lymphocytes and monocytes from healthy donors (with and without prior scabies sensitization) were stimulated with *S. scabiei* var. *canis* extract [47]. All donors exhibited an increased secretion of IL-10 and IFNγ in response to stimulation with scabies extract. A lack of IL-2 or IL-4 production suggested that regulatory T cells (Treg) cells were responsible for the generation of this cytokine profile. Likewise, PBMCs from individuals without scabies exposure produced increased IL-10 when stimulated with cysteine protease Yv5032C08 [46]. The upregulated production of IL-10 is significant because this cytokine depresses the inflammatory and immune responses in humans and thus may contribute to the delayed manifestation of clinical symptoms characteristic of early scabies infections.

While burrowing in the lower epidermis of the skin, scabies mites ingest plasma from the host that contains host antibody [27] and other plasma components including serine proteases, complement and other enzymes that could damage the mite gut lining that is crucial to the digestion and absorption of nutrients. The mite gut secretes, among other things, catalytically inactive serine protease paralogs (SMIPPs) [48, 49]. SMIPPs can inhibit complement activity by binding C1q, mannose binding lectin, and properdin in the three complement pathways and thus they protect the mite gut lining from complement attack and damage [48–50]. In addition, serine protease inhibitors (serpins) also called SMS (for scabies mite serpins) are localized in the mite gut and feces [51]. Two recombinant serpins, SMS B3 and SMS B4, can inefficiently inhibit mammalian serine proteases but not mite serine or cysteine proteases. Mika et al. [51] showed that these mite serpins could bind to several plasma proteins in the human classical, alternative, and lectin complement systems and also possibly block or downregulate the effectiveness of all three complement systems. Hence, these mites have evolved a variety of molecules allowing them to protect themselves from the host’s complement defense mechanisms.

Secondary bacterial infections often develop in scabies lesions and accompany a scabies infection [52–54]. The SMIPP and SMS complement inhibitors in mite feces may contribute to the survival and growth of bacteria in the skin of the host [52, 53]. Mites deposit fecal pellets in the burrow behind them as they feed and lengthen the burrow. Thus, the expelled fecal pellets containing the SMIPPs and SMSs that are deposited in the skin may inhibit the host complement activity in the skin/plasma surrounding the mites and inhibit the complement attack on bacteria in the lesion as well and thus promote the bacterial colonization that often accompanies a chronic scabies infection.

Collectively, all of these complex interactions likely delay the immune and inflammatory response early in an infection while a mite population is being established. This delay is eventually over-ridden once the mite population reaches a threshold and the inflammatory reaction occurs.

### Response to scabies molecules by cells in the primary lymphoid tissue of a host

Studies in mice show that mRNA expression for intercellular adhesion molecule-1 (ICAM-1), ICAM-2, VCAM-1 and L-selectin and the TNF receptors I and II (TNF RI and TNF RII) in splenic tissue was reduced in mice exposed to live *S. scabiei* var. *canis* (primary response) [55]. In contrast, mice first immunized with a *S. scabiei* var. *canis* extract and then exposed to live scabies mites (secondary response) did increase gene expression for ICAM-1, ICAM-2, TNF RI and RII. This suggests that immunization with relevant scabies mite antigens would be beneficial in reducing the mites’ ability to down-modulate the immune response and thus may help protect against scabies.

Also, expression of mRNA by splenic B- and T-cells of mice exposed to live scabies mites exhibited reduced expression of B7–2/CD86, CD4, CD8 and CD45 [55]. These membrane molecules play a key role in the interaction of B-cells and Th2 helper cells and between Th1 helper cells and cytotoxic T-cells in an immune reaction and suggests scabies mites produce something that can depress the immune reaction in lymphoid tissue.

The T-helper cell profile of splenocytes and lymph node cells were also determined in the mice [56]. Mice exposed to live scabies mites had increased IL-4 production by lymph node cells and of IFNγ by splenocytes. In contrast, mice immunized with mite extract prior to infestation shifted the response of lymph node cells from a Th2 (IL-4) response to a Th1 (INFγ) response.

### Scabies mite genomics

The development of molecular techniques has opened new avenues of research to understand the biology of scabies mites and the interactions between the mites and their hosts. The genomes of *S. scabiei* var. *canis* [57], var. *hominis* [58, 59] and var. *suis* [58, 59] have now been sequenced and these data are available for use as tools for clarifying the phylogenetic relationships and host preference of scabies mites parasitizing various mammals, identifying proteins involved in modulating the host protective responses, and identifying genes coding for proteins that are antigenic and could be candidates for inclusion in a vaccine or diagnostic blood test.

An annotated *S. scabiei* var. *canis* draft genome has been produced and is available in the NCBI and VectorBase databases [57] while the var. *hominis* and var. *suis* genomic data are available in the NCBI and GigaScience repositories [58, 59]. Generally, the genomes of the three strains are very similar. The var. *canis* genome contained genes for 10,644 predicted proteins [57] while the var. *hominis* genome contained 13,226 putative coding sequences [58]. By comparison, both scabies mite genomes have fewer genes than were predicted for the only four other acari with annotated genomes to date. The dust mite *Dermatophagoides farinae* has 16,376 predicted proteins while the spider mite *Tetranychus urticae* has 18,423, the deer tick *Ixodes scapularis* has 20,474, and the mesostigmatid mite *Metaseiulus occidentalis* has 11,444 [60–63]. The *S. scabiei* var. *canis* genome contains 3351 predicted proteins that are not orthologs of proteins present in the other mites [57]. This is important because a confounding factor in identifying proteins to include in a vaccine or for use in a blood test to specifically identify a scabies infection is the cross-reactivity between many scabies mite proteins and the ubiquitous dust mite antigens.

Using the predicted proteome, it was possible to definitively identify >150 proteins present in water soluble and insoluble extracts of whole mite bodies using MALDI-TOF/TOF mass spectrometry [64]. Many of these proteins are recognized by IgM and/or IgG in the serum of patients with ordinary scabies and may be candidates for inclusion in a diagnostic test for this disease. Others do not bind circulating antibody and may be involved in modulating the host’s immune defenses to protect the mite.

The genome has been also mined to identify predicted proteins that may be associated with scabies mite-host interactions and selected essential biological processes [65].

### Genetic diversity of scabies mites

One of the first molecular biology studies of *S. scabiei* identified multiple nucleotide sequence microsatellites in a partial genomic library of *S. scabiei* var*. hominis* [66]. Among these, the dinucleotide GA (guanine adenine) repeat was relatively abundant with repeat lengths up to 20. It was suggested that selected microsatellite patterns might be used to differentiate among various populations of mites.

Using the DNA fingerprinting technique reported above on scabies mites from humans and dogs provided some interesting insights into the genetic variability of *S. scabiei* both within and between host species [67]. It was found that patterns of microsatellite nucleotide repeats from mites collected from humans in northern Australia and in Panama differed significantly from DNA extracted from mites collected from dogs (some from the same locations) suggesting that the two mite strains have different transmission cycles, even for infected humans and dogs living in the same household. Thus, scabies mites from dogs are not likely the source of permanent scabies infections in humans at least in northwestern Australia. Comparing scabies mites from dogs within a community, they found there may or may not be significant genotypic differences. The latter findings suggested significant subpopulations of *S. scabiei* in dogs. When they compared various human isolates, there was also significant genetic variability between scabies mites from different households within communities in Australia but little genetic differentiation between scabies mites from individuals within the same household. The latter finding suggested a common source of scabies mites for the infected individuals living in the same household. Likewise, genetic differences existed between scabies mites from humans in communities in Australia and scabies mites from humans in Panama. Taken together, these data suggest there are subpopulations of scabies mites within a host species and this raises the possibility of multiple species of scabies mites within humans and other host populations. This concept was more recently supported by research in China suggesting that there are many different strains (species) of scabies mites that parasitize humans [68]. Based on the mitochondrial cytochrome *c* oxidase subunit 1 (mtDNA *cox*1) gene, the *Sarcoptes* from humans in Australia, Panama, and 2 populations in China were reported to represent 4 different species of *Sarcoptes* [68].

Studies analyzing ribosomal second internal transcribed spacer DNA (rDNA ITS2) and mitrochrondrial 16S DNA (mtDNA 16S) have found no interspecific differences among *Sarcoptes* mites collected from different host species [69–72]. However, other studies that analyzed mtDNA 16S and mtDNA *cox*1 and rDNA ITS2 of scabies mites from different animal hosts found they exhibited differences [73, 74]. A similar analysis by Zhao et al. [68] identified scabies mites from humans and scabies mites from dogs in China as distinct *Sarcoptes* populations but humans could be infected with *Sarcoptes* from dogs [68]. However, they also concluded that based on the 317-bp mtDNA *cox*1 gene, scabies mites from buffalo, rabbits, sheep, wombats, wallabys, pigs, chimpanzees and dogs belong to the same species and that the scabies mites from humans are a separate species from the animal species [68].

Likewise, Andriantsoanirina et al. [75] by analyzing the mitrochondrial gene coding for 12S-rRNA of mites from different hosts concluded that mites from wombats, dogs, and humans do not diverge phylogenetically and that scabies in wombats in Australia likely came from humans and/or their animals. This is in contrast to an earlier study that found that mites collected from wombats did not cluster with those collected from humans or dogs [67].

Another study using *cox*1 by this same group [76] has also suggested that human scabies mites do not constitute a single homogeneous population. Furthermore, using *cox*1 gene polymorphisms of mites from humans in France, dogs, and data from GenBank they concluded that scabies mites from humans were distributed into three genetically distinct and isolated clades (A, B and C) and that dog and human mites were not genetically different. Furthermore, Clade C contained scabies mites from humans and 12 other host species (dog, rabbit, chimpanzee, pig, sheep, water buffalo, cattle, wombat, wallaby, raccoon dog, serow and marten) and that gene flow occurs between mites from different hosts.

A study by Mofiz et al. [59] reported that the mitochondrial genome sequences of mites collected from humans, pigs and dogs were very similar. They then identified single nucleotide polymorphisims (SNPs) that were used to detect the presence of six haplotypes among the var. *hominis* and var. *suis* samples. The data indicated that the mites from one human patient appeared essentially clonal while the mites collected from another patient showed highly divergent haplotypes, including ones that clustered with the haplotypes present in mites from pigs.

Investigations using microsatellite DNA to study the genetic diversity between sympatric and non-sympatric hosts and their scabies mite parasites have provided some additional interesting indirect information about host specificity and genetic diversity [74, 77–79]. Generally, microsatellite analysis suggests that there is only one highly variable species of *S. scabiei* [74, 77–79]. However, microsatellite genotyping of individual *S. scabiei* mites collected in three European countries from 15 wild mammal populations belonging to 10 host species clustered into 3 groups (host-taxon derived): herbivore-derived, carnivore-derived, and omnivore-derived [80]. Generally, the microsatellite analysis results showed a lack of gene flow between these groups but gene flow could occur within a group. However, mange-free mammal species could live within the same geographical area as mangy animals so physiological, immune defenses and other properties of the host prevent the transfer and colonization between different sympatric potential host species.

It is possible that genetic make-up (allele presence and frequency) of a scabies strain may change (drift) over time. Alasaad et al. [78] using one multiplex of 9 microsatellite loci further investigated the temporal genetic diversity in populations of mites over 11 years and found little change in genetic diversity of *S. scabiei* among the sympatric wild mammal populations with *S. scabiei* in populations from Pyrenean chamois in Asturia (Spain) red deer, roe deer, and red foxes.

It is unclear just how host specific *S. scabiei* collected from different mammalian hosts are and if there are multiple species of *Sarcoptes* both within and between host species. Cross-breeding studies are not possible and cross-infectivity studies are very limited. Results of molecular studies provide some interesting insights into this question but also inconclusive answers because different nuclear, ribosomal and mitochondrial genes were used in these studies.

### Cross-host species transfers

Mites from scabies infected dogs can establish permanent infections on domestic rabbits and these mites can re-infect dogs [9]. This suggests that roaming/outdoor domestic dogs and wild canines that prey on wild rabbits with scabies may acquire scabies from these hosts and thus scabies is cross-infective between some canine species and rabbit hosts. Similarly, wild chamois with scabies have been shown to infect domestic goats and vice versa and red foxes with scabies can infect domestic dogs [81]. Analysis of nine microsatellite markers suggests that raccoons in Germany may acquire scabies from scabies infected red foxes [82].

In another analysis using a multiplex of 9 microsatellite markers, Oleaga et al. [83] compared genetic diversity among scabies mites collected from the sympatric Iberian wolf, chamois, red deer, roe deer and red fox. Mites from the Iberian wolf had the greatest genetic diversity. This suggested that prey-to-predator transfer may modify the host-taxa relationship. It would seem logical that prey-to-predator transfer and coevolution of the parasite in these predator-prey associations are important in the transfer of *S. scabiei* between hosts and thus the sharing of a common strain of *S. scabiei* between prey and predator is very likely. Gakuya et al. [84, 85] suggested a prey-to-predator transfer between wild felids and ungulates in Africa.

### Diagnosis and blood test for scabies

It is not difficult to diagnose scabies for human patients with crusted scabies and domestic and wild animals with advanced scabies (including dogs, foxes, coyotes, deer, goats, chamois, rabbits and others) because they harbor thousands of mites that are easily recovered by skin scraping. However, early diagnosis of ordinary scabies mite infections is very difficult in humans and other mammals because early infection involves very few mites and no or minimal symptoms are exhibited for many weeks. It is reported that the number of adult mites found on a human with ordinary scabies is low and fewer than 15 (mean 11.2) [86]. This number is based on the examination of about 900 adult patients. Early scabies infections in canines and other animals as well harbor few mites. This makes a positive diagnosis of scabies very difficult by skin scrape. Furthermore, diagnosis of scabies is also complicated because the clinical manifestations of a scabies infection mimic those of other skin diseases (atopic dermatitis, eczema, psoriasis, diaper rash, insect bites, poison ivy, etc.) and skin conditions caused by irritating agents (soaps/detergents, lotions, fragrances, metals, latex, rubber and other chemicals). A presumptive diagnosis may be made based on clinical symptoms such as intense itch, rash and erythematous patches but a definitive diagnosis is made based on microscopic demonstration of mites, their eggs and/or fecal pellets, and burrows in the epidermis that are removed from the skin by scraping with a scalpel. However, finding and scraping scabietic lesions can be difficult. Thus, there has been much interest in developing a diagnostic blood test for scabies in both humans and animals. It is clear that scabies mite infections induce a humoral response by the host that results in circulating antibody (which may or may not be protective). So, a blood test is feasible but still requires considerable research in order to achieve close to 100% specificity and sensitivity. A blood test based on detecting serum antibody to scabies mite antigens would be a great benefit for the early diagnosis of scabies when recovering mite material in a skin scrape is difficult.

A blood test to diagnose a scabies infection requires that appropriate scabies mite antigens be identified and produced and that the appropriate host antibody isotype and specificity be identified. Adequate quantities of scabies mites cannot be mass cultured in vivo or in vitro to obtain large amounts of antigen for use in a diagnostic test or for vaccine development to protect against infection. Because of the scarcity of mite material, the antigen molecules needed for both a diagnostic test and for vaccine development will likely depend on identification and production of recombinant molecules that are recognized by circulating antibody that the host builds in response to an active scabies infestation. Some recombinant molecules have been produced and screened for this purpose in dogs, pigs, and rabbits. Progress is being made but the key recombinants are yet to be identified. One of the problems that must be overcome in developing this test is the high level of cross-reactivity between antigens from scabies mites and antigens from the related ubiquitous house dust mites. A high percentage of individuals worldwide are allergic to house dust mites and produce IgE and IgG antibody to their allergens and antigens, respectively. A serological test based on detecting scabies mite specific IgE would not be good for detecting scabies mite infections because, like for house dust mite allergy, not all patients are predisposed to produce IgE to them and thus they are not allergic to their antigens. A patient with scabies may or may not be allergic to house dust mites but most patients with scabies appears to be sensitized to house dust mites [87].

As previously mentioned, the antibody isotype used for detecting scabies mite antigen is important. Hosts likely produce IgM antibody to mite antigen before switching to IgG. Thus, early diagnosis likely should be based on detecting IgM or both IgM and IgG in the blood of scabietic patients. A preliminary study supports this concept. A study by Arlian et al. [87] found that 45.1%, 27.5% and 73.6% of 91 patients with ordinary scabies had serum antibody Ig, IgG and IgM to scabies mite antigens, respectively. Only 2.2% had IgE directed at scabies mite antigens. However, as aluded to earlier, cross-reactivity and sensitization to house dust mites complicates the picture. Of the 91 scabetic patients, 84.6%, 91.2% and 86.8% had IgG to *D. farinae*, *D. pteronyssinus*, and *E. maynei*, respectively. Likewise, 75.8%, 83.5%, and 84.6% had IgM directed at these three mite species, respectively. But only 2–3% had IgE directed to any of the four mite species tested (*S. scabiei*, *D. farinae*, *D. pteronyssinus* and *E. maynei*). Many antigens from house dust mites have cross-reactive epitopes with antigens from scabies mites [88–92]. Thus, the protein molecules used for detection of scabies antibody should not be cross-reactive with any antigenic proteins or peptides from house dust mites. It is likely that a diagnostic blood test will need to contain a cocktail of scabies mite proteins/peptides [87]. If indeed there are multiple strains of *S. scabiei* that infects humans in different geographical areas of the world as has been suggested [68], this combination of antigens and the blood antibody profile may need to be developed and tailored to particular areas/regions of the world.

In contrast to patients with ordinary scabies, it appears most patients with crusted scabies have IgE directed at scabies mite antigens. A study by Arlian et al. [93] investigated the IgE and IgG profiles of patients with ordinary and crusted scabies. Immunoblotting found that 6 of 6 patients with crusted scabies had serum IgE that recognized from 11 to 21 proteins/peptides in a whole body *S. scabiei* var. *canis* extract separated by SDS-PAGE [93]. In contrast, only 3 of 7 patients with ordinary scabies had IgE and only 2 of 7 had IgG that recognized protein bands and their binding was weaker, indicating much lower titers. Walton et al. [46] also demonstrated that patients with crusted scabies had significantly higher levels of serum IgE that recognized 4 recombinant *S. scabiei* var. *hominis* proteins with homology to dust mite allergens than did patients with the ordinary form of the disease.

Serological enzyme-linked immunosorbent assays (ELISAs) for the diagnosis of scabies have been developed and evaluated for use in several different host species. These tests showed varied success and the results depended on the degree and duration of an infestation and the target antigen used.

The first studies used aqueous antigenic extracts made from *S. scabiei* whole mite bodies (Table 3). Production of these extracts required the tedious task of collecting mites from various hosts. Wells in ELISA plates were coated with antigen in these extracts. In some cases, the mite antigen was matched with the strain of mite responsible for the infection (e.g. fox mite antigen and infected foxes, pig mite antigen and infected pigs). In other cases, mite antigen from one host species was used to detect antibody in the serum from a different host species infected with scabies. For example, fox mite antigen was used to detect antibodies in the serum from infected pigs and dog scabies mite antigen was used to detect antibody in serum from scabies infected humans. Thus, the results relied on cross-reaction or common antigens between scabies mites from different host species. Laboratory studies using crossed immunoelectrophoresis and immunoblotting have directly demonstrated cross-reactivity between antigens of scabies mites from pigs (var. *suis*), dogs (var. *canis*), and humans (var. *hominis*) and that these mites are the sources of cross-reacting antigens or epitopes which supports the approach of using antigen derived from one mite strain to detect antibody in the serum of a host infected with a different strain [94].

**Table 3:** Characteristics of published ELISAs using crude mite extract as the target antigen to detect antibodies circulating in serum of scabietic hosts

Target antigen		Host species	Detecting antibodies		Sensitivity (%)	Specificity (%)	Reference
Mite variety	Extract		Primary	Conjugate			
*canis* (on Rb)	Aq Hmgt	rabbit (*canis*)		HRP-Go × Rb Ig			[116]
*canis* (on Rb)	Aq Hmgt	rabbit (*canis*)		HRP-Go × Rb Ig			[90]
*canis* (on Rb)	Aq Hmgt	rabbit (*canis*)		HRP-Go × Rb IgM			[90]
*canis* (on Rb)	Aq Hmgt	dog		HRP-Go × Dog Ig			[117]
*canis* (on Rb)	Aq Hmgt	dog		HRP-Go × Dog IgM			[117]
*canis* (on Rb)	Aq Hmgt	dog		HRP-Go × Dog IgG1			[117]
*canis* (on Rb)	Aq Hmgt	dog		HRP-Go × Dog IgG2			[117]
*canis* (on Rb)	Aq Hmgt	human		HRP-Go × Hu Ig			[127]
*canis* (on Rb)	Aq Hmgt	human	BT-Go × Hu Ig	HRP-SA	45		[87]
*canis* (on Rb)	Aq Hmgt	human	BT-Mu × Hu IgD	HRP-SA	0		[87]
*canis* (on Rb)	Aq Hmgt	human	BT-Mu × Hu IgE	HRP-SA	2		[87]
*canis* (on Rb)	Aq Hmgt	human	BT-Go × Hu IgG	HRP-SA	28		[87]
*canis* (on Rb)	Aq Hmgt	human	BT-Go × Hu IgM	HRP-SA	74		[87]
*canis*		ibex		HRP-Dnky × Go IgG	33	89	[128]
*caprae*	PBS Hmgt	goat		HRP-Dnky × Go IgG			[118]
*caprae*	PBS Hmgt	sheep	Mu × Sh IgG	HRP-SA	90		[129]
*cuniculi*	PBS Hmgt	mice		HRP-Go × Mu			[126]
*ovis*	PBS Hmgt	sheep	BT-Mu × Sh IgG; BT-Go × Mu IgG	HRP-SA	88	96	[102]
*suis*		pig			100		[96]
*suis*	Aq Ext	pig		HRP-Rb × Pig Ig	78	97	[130]
*suis*	Aq Ext	pig		HRP-Rb × Pig IgG-Fc	54	94	[98]
*suis*	Aq Ext	pig		HRP-Rb × Pig IgG-Fc	92	99	[98]
*suis*	Aq Ext	pig		HRP-Rb × Pig?	73	81	[131]
*suis*	Aq Ext	pig^1^		HRP-Rb × Pig?	71	77	[131]
*suis*		pig			89		[97]
*suis*	PBS Hmgt	pig		HRP-Rb x Pig IgG	100	100	[132]
*suis*		wild boar			75	80	[133]
*suis*		ibex	BT-rProtein G	HRP-avidin	93	94	[128]
*vulpes*	Aq Ext	red fox^2^	Mu x Dog IgG	HRP-Rb × Mu Ig	95	~100	[103]
*vulpes*	Aq Ext	red fox	Mu x Dog IgG	HRP-Rb × Mu Ig	95	83	[134]
*vulpes*	Aq Ext	red fox^2^	Mu x Dog IgG	HRP-Rb × Mu Ig	95–100		[135]
*vulpes*	Aq Ext	wolf	Mu x Dog IgG	HRP-Rb × Mu Ig			[136]
*vulpes*		dog		AP-Go × Dog IgG	84	90	[137]
*vulpes*	PBS Hmgt	dog		HRP-Mu × Dog IgG			[104]
*vulpes*	PBS Hmgt	dog		HRP-Mu × Dog IgG	83	92	[138]
*vulpes*	Aq sonicate	chamois	BT-rProtein G	HRP-avidin	93	97	[99]
*vulpes*	Aq sonicate	ibex	BT-rProtein G	HRP-avidin			[139]
*vulpes*	PBS Hmgt	pig		HRP-Rb × Pig Ig			[140]
*vulpes*	PBS Hmgt	pig		HRP-Go × Pig Ig	50–80	≥ 98	[95]
*vulpes*	PBS Hmgt	pig			30		[97]
*vulpes*	PBS Hmgt	pig			53		[97]
*vulpes*	PBS Hmgt	pig			70		[97]
*vulpes*	PBS Hmgt	pig			0–100		[96]
*vulpes*	PBS Hmgt	pig			100		[96]
*vulpes*	PBS Hmgt	human		HRP-Go × Hu IgG	48	100	[105]

All ELISAs tested serum except pig^1^ that tested meat juice and red fox^2^ that tested body fluids

*Abbreviations*: *AP* alkaline phosphatase, *Aq* Aqueous (presumably water), *BT* biotinylated, *Dnky* donkey, *Ext* extract, *Go* goat, *Hmgt* homogenate, *HRP* horseradish peroxidase, *Hu* human, *Mu* mouse, *PBS* phosphate buffered saline, *Rb* rabbit, *SA* streptavidin, *Sh* sheep, x = anti-;? = not reported

Hollanders et al. [95] used antigens in *S. scabiei* var. *vulpes* extract (whole mite body homogenate from fox mites) in an ELISA to detect antibody in the serum of documented scabies infected weaner, fattener and sow pigs. The analysis detected antibody in 80%, 77.8% and 50% of the weaners, fatteners and sows, respectively while more than 98% of the control pigs without scabies did not have detectable serum antibody to mite antigens.

Three commercial ELISAs (Checkit® Sarcoptest, Sarcoptes-ELISA 2001 PIG and Acar-Test P* ELISA) were used to detect IgG antibody in pigs experimentally infested with *S. scabiei* var. *suis* at various times as the infection progressed [96]. This controlled study showed that these ELISA systems could detect antibody in most of the infected pigs after 5 to 6 weeks of infection with scabies mites. A similar study used four different IgG ELISA kits to detect serum IgG to scabies mite antigen in naturally infected sow and guilt pigs [97]. One test kit used antigen from *S. scabiei* var. *suis* (pig mites) while the other three kits contained antigen from *S. scabiei* var. *vulpes* (fox mites). Serological positives ranged from 30% to 88.6% depending on the test used. However, skin scrapings were positive in only 48.6% of these pigs. Thus, some diagnostic serological tests can greatly improve diagnosis.

Van der Heijden et al. [98] also developed an Animal Health Service ELISA (AHS-ELISA) for the detection of scabies in pigs. The detecting antigen material was prepared from *S. scabiei* var. *suis* mites collected from sows with crusted mange. The AHS-ELISA detected serum antibody after 6 weeks in 5% of the pigs and in 74.2% of the pigs after 16 weeks with the infection. This illustrates that antibody responses are slow to develop or that the titer is low and below the detection limit of the technique during early scabies infections.

Scabies can be a major threat to wild chamois and Ibex in southern Europe [99–101]. Development of a serological test would aid resource managers to monitor infections in these animals. Rambozzi et al. [99] developed a biotin-avidin amplified ELISA (LAB-ELISA) using antigen from *S. scabiei* var. *vulpes* from red foxes (*Vulpes vulpes*) to detect antibodies in the sera of scabies infected chamois and distinguish them from healthy chamois. Using this test, 37 of 40 scabietic chamois were ELISA-positive while 101 of 104 sera collected from uninfected chamois were negative.

ELISA has been used to diagnose scabies in dogs as well. Lower et al. [99] evaluated an indirect ELISA (Imovet sarcoptes) in 19 dogs with confirmed scabies infections. The capture antigen in this test was *S. scabiei* var. *vulpes* from foxes and 84.2% of the dogs had detectable levels of IgG to the fox mite antigen while 89.2% of the control dogs with no history of scabies did not.

An ELISA using antigen from *S. scabiei* var. *ovis* has been used to detect serum antibody in sheep infected with var. *ovis* [102]. This ELISA diagnosed 87.6% of sheep with scabies while only 62.8% were positive by skin scraping.

Bornstein et al. [103] used ELISA plates coated with aqueous extract of antigens made from *S. scabiei* var. *vulpes* and secondary antibody raised against dog IgG to detect antibody in the blood-tinted body cavity liquids from red foxes. Presence of antibody to scabies mite antigen was found in 62 of 65 (95%) of red foxes that had clinical signs of sarcoptic mange.

An ELISA using a monoclonal anti-dog IgG has been used for detecting serum IgG directed at antigen from *S. scabiei* var. *vulpes* for the diagnosis in dogs infected with *S. scabies* var. *canis* mites [104]. Ten of 12 (83%) dogs with confirmed *S. scabiei* var. *canis* infection gave a positive ELISA result for *S. scabiei* var. *vulpes* antigens.

Haas et al. [105] evaluated the possibility of using *S. scabiei* var. *vulpes* antigen from fox mites in an ELISA format to diagnosis human scabies. ELISA plates were coated with fox mite antigen and then reacted with serum from scabietic patients. Sera from 41 human patients with confirmed *S. scabiei* var. *hominis* were screened by ELISA to detect IgG antibody. Forty eight percent of the scabietic patients had serum IgG that clearly recognized var. *vulpes* antigen and 17% were borderline positive. However, most patients that did not have IgG that recognized *S. scabiei* var. *vulpes* had symptoms for less than 4 weeks and the assay did not test for IgM antibody binding. Thus, IgM antibody isotype, that is produced prior to the switch to IgG production, may have been better for detection of scabies infection as suggested by Arlian et al. [87].

More recent studies have focused on using recombinant molecules to develop scabies mite diagnostic ELISAs (Table 4). Iberian red deer (*Cervus elaphus hispanicus*) in European countries can be infected with sarcoptic mange [106]. Oleaga et al. [106] used an ELISA to evaluate the presence of scabies mite-specific antibody in serum samples collected from 327 red deer without obvious sarcoptic lesions. This ELISA used the scabies mite recombinant antigen Ssλ20ΔB3 isolated from a *S. scabiei* var. *hominis* expression library [107]. It was recognized by antibody in serum from 6 of 8 scabietic red deer.

**Table 4:** Characteristics of published ELISAs using recombinant proteins as the target antigen to detect antibodies circulating in serum of scabietic hosts

Target antigen		Host species	Detecting antibodies		Sensitivity (%)	Specificity (%)	Reference
Mite variety	rProtein		Primary	Conjugate			
*cunniculi*	tropomyosin	rabbit		HRP-Go × Rb IgG			[141]
*cunniculi*	SsTPx	rabbit		HRP-Go × Rb IgG	100	100	[142]
*cunniculi*	cofilin	rabbit		HRP-Go × Rb IgG	83	88	[143]
*cunniculi*	calmodulin	rabbit		HRP-Go × Rb IgG	88	23	[109]
*hominis*	rSar s 14.3	pig		HRP-Rb × Pig IgG	100	100	[132]
*hominis*	rSar s 14.3	human	BT-Mu × Hu IgE	Eu-SA	100	94	[144]
*hominis*	Ssλ20ΔB3	chamois		HRP-Protein G	100	97	[107]
*hominis*	Ssλ20ΔB3	deer		HRP-Protein G	100	97	[107]
*hominis*	Ssλ20ΔB3	deer		HRP-Protein G	75	97	[106]
*hominis*	Ssλ20ΔB3	ibex		HRP-Protein G	58	78	[128]
*hominis*	Ssλ20ΔB3	pig		HRP-? × Pig IgG	40	86	[145]
*hominis*	Ssλ20ΔB3	pig		HRP-? × Pig IgG	62	83	[145]
*hominis*	Ssλ20ΔB3	rabbit		HRP-Protein A		100	[108]
*hominis*	Ssλ20ΔB3	rabbit		HRP-Protein A	95	97	[146]
*hominis*	Ssλ20ΔB3	wolf		HRP-Protein A	75	88	[147]
*hominis*	rSsGST01	human		AP-? × Hu IgV			[148]
*hominis*	rSj26GST	human		AP-? × Hu IgV			[148]
*suis*	SsE2	human		HRP-Go × Hu?	25		[111]
*suis*	SsE3	human		HRP-Go × Hu?	85		[111]
*suis*	SsE5	human		HRP-Go × Hu?	60		[111]
*suis*	SsE7	human		HRP-Go × Hu?	25		[111]
*suis*	SsE8	human		HRP-Go × Hu?	15		[111]
*suis*	SsE9	human		HRP-Go × Hu?	0		[111]
*suis*	SsE2	pig		HRP-Go × Pig?	4.8		[111]
*suis*	SsE3	pig		HRP-Go × Pig?	4.8		[111]
*suis*	SsE5	pig		HRP-Go × Pig?	4.8		[111]
*suis*	SsE7	pig		HRP-Go × Pig?	61.9		[111]
*suis*	SsE8	pig		HRP-Go × Pig?	4.8		[111]
*suis*	SsE9	pig		HRP-Go × Pig?	14.3		[111]

*Abbreviations*: *AP* alkaline phosphatase, *BT*, biotinylated, *Eu* europium, *Go* goat, *HRP* horseradish peroxidase, *Hu* human, *Mu* mouse, *Rb* rabbit, *SA* streptavidin, *V* various isotypes, x = anti-;? = not reported

In another study, the recombinant Ssλ20ΔB3 derived from *S. scabiei* var. *hominis* was shown by ELISA to detect antibody in 7 chamois (*Rupicapra rupicapra*) and 3 deer with confirmed scabies infections [107]. Of the healthy unexposed scabies-free animals, only 1 of 33 chamois and 1 of 33 deer were immunopositive. While the sample size of infected animals was small, the study demonstrated that a protein from human scabies mites did identify antibody to scabies mite antigens in the blood of two different species of animal hosts.

The wild European rabbit (*Oryctolagus cuniculus*) occurs in north-eastern Mediterranean Spain and serves as prey for many predators as well as game for hunters [108]. *Sarcoptes scabiei* parasitizes some populations of these rabbits and there is concern that the disease can significantly affect the rabbit population and that it might be transmitted to other wildlife. Thus, Millan et al. [108] used an ELISA based on the recombinant human scabies mite antigen Ssλ20ΔB3 to survey for the seroprevalence of scabies antibodies in rabbits in selected provinces or islands of Spain. Most of the sera analyzed were from rabbits shot by hunters. The results demonstrated that the human mite antigen could be used to detect scabies in wild rabbits and that exposure to *S. scabiei* was widespread among wild rabbits across Spain.

He et al. [109] used a recombinant calmodulin cloned from RNA extracted from *S. scabiei* that were collected from rabbits to develop an ELISA to detect the presence of *S. scabiei-*specific antibodies in the serum of rabbits infested with either *S. scabiei* or with *Psoroptes cunniculi,* another ectoparasite of rabbits. Although this assay had a sensitivity of 88%, its specificity was only 23% due to the cross-reactivity of the highly conserved calmodulin from scabies mites with this protein from *P. cunniculi*.

Mattsson et al. [110] used a recombinant 17.5 kDa fragment of var. *vulpes* paramyosin (miniaturized paramyosin) to detect antibody in the sera of scabies infected dogs and pigs by immunoblotting. This molecule was recognized by antibody from immunized rabbits but was not easily detected by antibody in the serum of scabies infected dogs and pigs.

Collectively, these studies demonstrate that there is enough cross-reactivity between scabies mite antigens from different hosts that natural or recombinant *S. scabiei* antigen from one host species can be used to detect serum antibody in a host infected with another strain of scabies mites. Also, these studies show the variability and difficulty in detecting serum antibody in naturally infected animals when the history of the infection is unknown. Key questions that impact the usefulness of a blood test for scabies are (i) how long does it take after an infection is initiated before antibody can be detected in serum, and (ii) what antibody isotype should this test detect (e.g. IgM, IgG, IgE, etc.)? These are factors that are yet to be determined but an early detection should probably look for IgM since this isotype is produced early in a humoral response. Also, should the blood test be designed to detect serum antibody directed at a single antigen or will it need to contain a cocktail of antigens to detect serum antibodies built to multiple antigens or to a specific profile of antigens? For instance, in the Kuhn study [111], might mixing target antigens SsE3 and SsE5 have increased sensitivity to 100% (Table 4)? These will be important factors to consider in the development of a blood test for scabies infections.

The emergence of molecular techniques coupled with the availability of mite genomic sequences provides the opportunity for the development of alternative diagnostic methods. Angelone-Alasaad et al. [112] designed two PCR-based methods for scabies diagnosis based on the amplification of 135 bp of the mitochondrial 16S rDNA. Both methods were successfully evaluated on scabies mites collected from 23 host species. The use of PCR-based technology is promising but still requires that mite material be recovered in a skin scrape, which remains a difficult task.

### Vaccination against scabies mite infestation

Acaricides are available for treatment of scabies but significance resistance to these acaricides has developed and thus treatment failures occur [113–115]. Additionally, these chemicals have known and unknown toxicity effects to humans and animals. Thus, vaccination for protection against infection by scabies mites is an attractive alternative to the currently available chemotherapies.

Vaccination against *S. scabiei* mites is a realistic goal for several reasons. Scabies mites induce both innate and adaptive immune responses in the parasitized host. The adaptive response involves production of IgM, IgG and in some human hosts IgE and IgA antibody isotypes to antigens the mites release in the epidermis as they burrow. The mites ingest serum antibody as they burrow and feed in the lower epidermis. Several lines of evidence suggest that serum is a component of their diet. Systemic acaricides, such as ivermectin, kill mites in the skin, presumably because it is ingested by the mites. A study using a fluorescently labeled antibody showed that host antibody bound to the gut lining of live mites removed from a host [27]. Host antibody that binds to molecules of the gut cells and digestive enzymes produced by these cells that are crucial for digestion and absorption of nutrients may block these processes and thus prevent survival of the mite. Likewise, host antibody directed at molecules from the mite that are crucial to its suppressing the host’s protective responses would hinder the mite’s ability to survive and establish a population in the host skin because the host could now mount a successful protective response.

Other lines of evidence suggest that vaccination may induce protection against scabies mites. Host antibody titers were found to develop more rapidly and with greater intensity during a second infection compared to an initial (primary) infection with scabies mites [116]. Animals that recovered from a scabies mite infection exhibit reduced levels of mites upon a subsequent reinfection [11, 116–119]. Arlian et al. [117] found that 8 dogs cured of *S. scabiei* and then reinfested, expressed protective immunity. Seven of the 8 dogs developed short-term infections that completely disappeared in time without any treatment. Finally, rabbits immunized with a whole body extract of *S. scabiei* var. *canis* produced antibodies to more antigens than rabbits infected with this mite [120].

The results of a few vaccination trials have been published. A study by Tarigan et al. [118] evaluated the protective effect of vaccinating goats with an extract made from whole bodies of scabies mites collected from infected goats and several fractions of this extract prepared by anion exchange chromatography. They found no difference in the severity of skin lesions between immunized and non-immunized goats at several times post-experimental infection although previously infected animals did exhibit protective immunity.

Harumal et al. [121] investigated the protective effect of immunizing rabbits with the recombinant antigens Ssag1 and Ssag2 (from var. *hominis* mites). Immunized rabbits produced antibody to both antigens however, the rabbits did not show any protective immunity or reduction in the mite numbers. The Ssag 1 and Ssag 2 molecules were located around internal organs and the cuticle of the scabies mite and in eggs. Apparently, these protein fragments are not accessible to antibody or antibody binding to them did not inhibit any vital function key to mite survival.

Casias et al. [122] vaccinated rabbits with a mix of the recombinant antigens Ssλ20ΔB3 and GST-Ssλ15 derived from *S. scabiei* var. *hominis* mites. These rabbits were then challenged with *S. scabiei* var. *cuniculi* (scabies mites from rabbits) and exhibited high specific IgG levels and increased levels of total IgE but were not protected against infestation by these mites.

Studies showed that the house dust mites *D. farinae* and *D. pteronyssinus* cross-react with antigens in extracts of *S. scabiei* [88, 89]. This suggested that vaccination with extracts of house dust mites may induce protection against *S. scabiei*. Large quantities of scabies mites are not available to make a vaccine for immunization purposes but house dust mites can be cultured in kilogram quantities. Immunization with a 50/50 mix of *D. farinae* and *D. pteronyssinus* extracts induced protective immunity to *S. scabies* var. *canis* infection in rabbits as evidenced by the marked reduction in parasite load compared to the unimmunized controls [90].

Other investigations provide proof of the principle that there can be protection against an ectoparasitic mite. Sheep and rabbits were vaccinated with the related scab mite *Psoroptes* sp. Partial immunity to infection with *P. cuniculi* (rabbit scab mite) developed after four immunizations with a whole body extract [123]. Vaccinated rabbits gave comparable protection compared to naturally infected rabbits. Likewise, vaccination of sheep with whole body extracts of *P. ovis* (sheep scab mite) prepared in saline or 1% Tween resulted in significant protection to infestation [124]. A subsequent investigation found that a specific fraction prepared by anion exchange chromatography of the parent extract gave greater protection than other fractions and than the parent extract itself [125]. However, a SDS-PAGE profile of the fraction showed it contained many proteins so the molecule(s) responsible for the increased protection could not be identified.

Gu et al. [126] investigated the immune response induced in mice by a *S. scabiei* var. *cuniculi* DNA vaccine encoding paramyosin. The DNA vaccine induced a humoral and cellular immune response characterized by higher levels of IgG, IgG1, IgG2a, IgE and IgM, increased secretion of IL-2, IL-4, IL-5 and IFNγ by splenocytes, and proliferation of lymphocytes in the spleen. Paramyosin is a common protein in many invertebrates and has a high homology between species. These experiments provide a basis for further study of a possible DNA vaccine to protect against scabies. It was not determined if this induced any protective immunity.

Taken together, all of these data clearly indicate that vaccination is a realistic possibility to protect human and animal populations against scabies mites. Many factors in addition to the antigen or antigen mix come into play when considering vaccine trials for scabies. Many of the reported immunization failures could be attributed to adjuvants, immunization schedule, antigen dose, and delivery method. The key antigen or cocktail of antigens have yet to be identified and produced by recombinant technology. In addition, all components of the immunization protocol will need to be determined.

### Conclusions

The disease scabies has been known for millennia. However, the lack of large quantities of scabies mites from hyper-infected hosts, in vitro culture methods or in vivo animal models have limited the types of studies that have been possible. Knowledge of the biology of scabies mites, host-parasite interactions, molecules the mites produce (antigens, non-antigens and immune-modulating), host-modulating abilities, host innate and adaptive immune responses and mite proteomics and genomics have increased tremendously during the last 30 years. Specifically, scabies mites from the rabbit model have allowed the systematic determination of the life-cycle and life-stage durations, and elucidated the host-seeking behavior and response to host stimuli, response to light, survival off the host and infectivity from fomites, skin penetration activity, water balance and nutrient procurement and identified factors in the skin that may influence the mite’s preferred site on the host body. Mites from humans and the rabbit and pig models have been used to determine that molecules from mites modulate the cytokine secretion from dermal keratinocytes, epidermal fibroblasts, lymphocytes, and endothelial cells of the microvasculature and expression of cell adhesion molecules from the later and block the three complement pathways. New molecular techniques now allow for genomic and proteomic characterization of scabies mites and the production of recombinant molecules. These later molecular tools now make the possibility for development of a much-needed diagnostic test for scabies and a vaccine to protect against scabies in vulnerable populations a reality. It is just a matter of time.

## Abbreviations

AP: Alkaline phosphatase
BT: Biotinylated
*cox*1: Cytochrome *c* oxidase subunit 1
CTACK: Cutaneous T cell-attracting chemokine
Dnky: Donkey
ELISA: Enzyme-linked immunosorbent assay
Eu: Europium
Ext: Extract
GA: Guanine adenine
G-CSF: Granulocyte colony stimulating factor
GM-CSF: Granulocyte-macrophage colony stimulating factor
Go: Goat
GROα: Growth related oncogene-α
Hmgt: Homogenate
HMVEC-D: Human dermal microvascular endothelial cells
HRP: Horseradish peroxidase
HSE: Human skin equivalents
Hu: Human
ICAM-1: Intercellular adhesion molecule-1
IFNγ: Interferon gamma
Ig: Immunoglobulin
IL: Interleukin
IL-1ra: IL-1 receptor agonist
LPS: Lipopolysaccharide
MALDI-TOF/TOF: Matrix-assisted laser desorption ionization time-of-flight
MCP-1: Monocyte chemoattractant protein-1
M-CSF: Macrophage colony stimulating factor/time-of-flight
mtDNA 16S: Mitrochrondrial 16S DNA
mtDNA *cox*1: Mitochondrial cytochrome *c* oxidase subunit 1 gene
Mu: Mouse
PBMC: Peripheral blood mononuclear cells
PBS: Phosphate buffered saline
Rb: Rabbit
rDNA ITS2: Second internal transcribed spacer of the ribosomal RNA gene
RH: Relative humidity
SA: Streptavidin
SDS-PAGE: Sodium dodecyl sulfate polyacrylamide gel electrophoresis
serpins: Serine protease inhibitors
Sh: Sheep
SMIPP: Scabies mite inactive protease paralog
SMS: Scabies mite serpins
SNP: Single nucleotide polymorphism
TGFα: Transforming growth factor-α
Th: T-helper cell
TNF RI: TNF receptor I
TNFα: Tumor necrosis factor-α
Treg: Regulatory T cells
TSLP: Thymic stromal lymphopoietin
VCAM-1: Vascular cell adhesion molecule-1
VEGF: Vascular endothelial growth factor
x: Anti-

## References

[1] Roncalli RA (1987). The history of scabies in veterinary and human medicine from biblical to modern times. Vet Parasitol.

[2] Friedman R (1947). The story of scabies.

[3] Zhang ZQ (2011). Animal biodiversity: an outline of higher-level classification and survey of taxonomic richness. Zootaxa.

[4] Bochkov AV (2010). A review of mammal-associated Psoroptidia (Acariformes: Astigmata). Acarina.

[5] Klompen H (1992). Phylogenetic relationships in the mite family Sarcoptidae (Acari: Astigmata). Misc Publ Univ Michigan Mus Zool.

[6] Fain A (1968). Etude de la variabilite de *Sarcoptes scabiei* avec une revision des Sarcoptidae. Acta Zool Pathol Antverp.

[7] Kummel BA, Estes SA, Arlian LG (1980). *Trixacarus caviae* infestation of guinea pigs. J Am Vet Med Assoc.

[8] Arlian LG, Runyan RA, Vyszenski-Moher DL (1988). Water balance and nutrient procurement of *Sarcoptes scabiei* var. *canis* (Acari: Sarcoptidae). J Med Entomol.

[9] Arlian LG, Runyan RA, Estes SA (1984). Cross infestivity of *Sarcoptes scabiei*. J Am Acad Dermatol.

[10] Mounsey K, Ho MF, Kelly A, Willis C, Pasay C, Kemp DJ (2010). A tractable experimental model for study of human and animal scabies. PLoS Negl Trop Dis.

[11] Mellanby K (1944). The development of symptoms, parasitic infection and immunity in human scabies. Parasitology.

[12] Heilesen B (1946). Studies on *Acarus scabiei* and scabies.

[13] Munro JW (1919). Report of scabies investigation. J R Army Med Corp.

[14] Van Neste D, Mrena E, Marchal G (1981). Life cycle of scabies mite (*Sarcoptes scabiei* var. *hominis*) studied by scanning electron microscopy (author's transl). Ann Dermatol Venereol.

[15] Arlian LG, Vyszenski-Moher DL (1988). Life cycle of *Sarcoptes scabiei* var. *canis*. J Parasitol.

[16] Ljunggren EL (2005). Molecular analysis of *Sarcoptes scabiei*.

[17] Arlian LG, Estes SA, Vyszenski-Moher DL (1988). Prevalence of *Sarcoptes scabiei* in the homes and nursing homes of scabietic patients. J Am Acad Dermatol.

[18] Arlian LG, Runyan RA, Sorlie LB, Estes SA (1984). Host-seeking behavior of *Sarcoptes scabiei*. J Am Acad Dermatol.

[19] Mellanby K, Johnson CG, Bartley WC, Brown P. Experiments on the survival and behavior of the itch mite *Sarcoptes scabiei* DeG var. *hominis*. Bull Entomol Res. 1942;33:267–71.

[20] Arlian LG, Runyan RA, Achar S, Estes SA (1984). Survival and infectivity of *Sarcoptes scabiei* var. *canis* and var. *hominis*. J Am Acad Dermatol.

[21] Wharton GW, Richards AG (1978). Water vapor exchange kinetics in insects and acarines. Annu Rev Entomol.

[22] Arlian LG, Veselica MM (1981). Reevaluation of the humidity requirements of the house dust mite *Dermatophagoides farinae* (Acari: Pyroglyphidae). J Med Entomol.

[23] Arlian LG (1992). Water balance and humidity requirements of house dust mites. Exp Appl Acarol.

[24] Van Neste D, Lachapelle JM (1981). Host-parasite relationships in hyperkeratotic (Norwegian) scabies: pathological and immunological findings. Br J Dermatol.

[25] Van Neste D (1984). Intraepidermal localization of scabies mites overlooked?. J Am Acad Dermatol.

[26] Estes SA, Kummel B, Arlian L (1983). Experimental canine scabies in humans. J Am Acad Dermatol.

[27] Rapp CM, Morgan MS, Arlian LG (2006). Presence of host immunoglobulin in the gut of *Sarcoptes scabiei* (Acari: Sarcoptidae). J Med Entomol.

[28] Morgan MS, Arlian LG (2006). Enzymatic activity in extracts of allergy-causing astigmatid mites. J Med Entomol.

[29] Arlian LG, Vyszenski-Moher DL (1995). Response of *Sarcoptes scabiei* var. *canis* (Acari: Sarcoptidae) to lipids of mammalian skin. J Med Entomol.

[30] Entrekin DL, Oliver JH (1982). Aggregation of the chicken mite, *Dermanyssus gallinae* (Acari: Dermanyssidae). J Med Entomol.

[31] Otieno DA, Hassanali A, Obenchain FA, Sternberg AG, R. (1985). Identification of guanine as an assembly pheromone of ticks. Insect Sci Appl.

[32] Sonenshine DE (1991). Tick pheremones. Anonymous biology of ticks.

[33] Sonenshine DE, Silverstein RM, West JR. Occurrence of sex attractant pheromone, 2,6-dichlorophenol, in relation to age and feeding in American dog tick, *Dermacentor variabilis* (Say) (Acari: Ixodidae). J Chem Ecol. 1984;10(1):95–100. 10.1007/BF00987646 24318231

[34] Sonenshine DE (1985). Pheromones and other semiochemicals of the acari. Annu Rev Entomol.

[35] Arlian LG, Vyszenski-Moher DL (1996). Responses of *Sarcoptes scabiei* (Acari: Sarcoptidae) to nitrogenous waste and phenolic compounds. J Med Entomol.

[36] Arlian LG, Morgan MS, Neal JS (2003). Modulation of cytokine expression in human keratinocytes and fibroblasts by extracts of scabies mites. Am J Trop Med Hyg.

[37] Mullins JS, Arlian LG, Morgan MS (2009). Extracts of *Sarcoptes scabiei* de Geer downmodulate secretion of IL-8 by skin keratinocytes and fibroblasts and of GM-CSF by fibroblasts in the presence of proinflammatory cytokines. J Med Entomol.

[38] Hajnicka V, Kocakova P, Slavikova M, Slovak M, Gasperik J, Fuchsberger N (2001). Anti-interleukin-8 activity of tick salivary gland extracts. Parasite Immunol.

[39] Deruaz M, Frauenschuh A, Alessandri AL, Dias JM, Coelho FM, Russo RC (2008). Ticks produce highly selective chemokine binding proteins with antiinflammatory activity. J Exp Med.

[40] Vancova I, Hajnicka V, Slovak M, Kocakova P, Paesen GC, Nuttall PA (2010). Evasin-3-like anti-chemokine activity in salivary gland extracts of ixodid ticks during blood-feeding: a new target for tick control. Parasite Immunol.

[41] Morgan MS, Arlian LG (2010). Response of human skin equivalents to *Sarcoptes scabiei*. J Med Entomol.

[42] Morgan MS, Arlian LG, Markey MP (2013). *Sarcoptes scabiei* mites modulate gene expression in human skin equivalents. PLoS One.

[43] Elder BL, Arlian LG, Morgan MS (2006). *Sarcoptes scabiei* (Acari: Sarcoptidae) mite extract modulates expression of cytokines and adhesion molecules by human dermal microvascular endothelial cells. J Med Entomol.

[44] Elder BL, Arlian LG, Morgan MS (2009). Modulation of human dermal microvascular endothelial cells by *Sarcoptes scabiei* in combination with proinflammatory cytokines, histamine, and lipid-derived biologic mediators. Cytokine.

[45] Arlian LG, Morgan MS, Neal JS (2004). Extracts of scabies mites (Sarcoptidae: *Sarcoptes scabiei*) modulate cytokine expression by human peripheral blood mononuclear cells and dendritic cells. J Med Entomol.

[46] Walton SF, Pizzutto S, Slender A, Viberg L, Holt D, Hales BJ (2010). Increased allergic immune response to *Sarcoptes scabiei* antigens in crusted *versus* ordinary scabies. Clin Vaccine Immunol.

[47] Arlian LG, Morgan MS, Paul CC (2006). Evidence that scabies mites (Acari: Sarcoptidae) influence production of interleukin-10 and the function of T-regulatory cells (Tr1) in humans. J Med Entomol.

[48] Holt DC, Fischer K, Allen GE, Wilson D, Wilson P, Slade R (2003). Mechanisms for a novel immune evasion strategy in the scabies mite *Sarcoptes scabiei*: a multigene family of inactivated serine proteases. J Invest Dermatol.

[49] Bergstrom FC, Reynolds S, Johnstone M, Pike RN, Buckle AM, Kemp DJ (2009). Scabies mite inactivated serine protease paralogs inhibit the human complement system. J Immunol.

[50] Fischer K, Langendorf CG, Irving JA, Reynolds S, Willis C, Beckham S (2009). Structural mechanisms of inactivation in scabies mite serine protease paralogues. J Mol Biol.

[51] Mika A, Reynolds SL, Mohlin FC, Willis C, Swe PM, Pickering DA (2012). Novel scabies mite serpins inhibit the three pathways of the human complement system. PLoS One.

[52] Mika A, Reynolds SL, Pickering D, McMillan D, Sriprakash KS, Kemp DJ (2012). Complement inhibitors from scabies mites promote streptococcal growth - a novel mechanism in infected epidermis?. PLoS Negl Trop Dis.

[53] Swe PM, Fischer K (2014). A scabies mite serpin interferes with complement-mediated neutrophil functions and promotes staphylococcal growth. PLoS Negl Trop Dis.

[54] Swe PM, Reynolds SL, Fischer K (2014). Parasitic scabies mites and associated bacteria joining forces against host complement defence. Parasite Immunol.

[55] Arlian LG, Fall N, Morgan MS (2007). *In vivo* evidence that *Sarcoptes scabiei* (Acari: Sarcoptidae) is the source of molecules that modulate splenic gene expression. J Med Entomol.

[56] Lalli PN, Morgan MS, Arlian LG (2004). Skewed Th1/Th2 immune response to *Sarcoptes scabiei*. J Parasitol.

[57] Rider SD, Morgan MS, Arlian LG (2015). Draft genome of the scabies mite. Parasit Vectors..

[58] Mofiz E, Holt DC, Seemann T, Currie BJ, Fischer K, Papenfuss AT (2016). Genomic resources and draft assemblies of the human and porcine varieties of scabies mites, *Sarcoptes scabiei* var. *hominis* and var. *suis*. Gigascience.

[59] Mofiz E, Seemann T, Bahlo M, Holt D, Currie BJ, Fischer K (2016). Mitochondrial genome sequence of the scabies mite provides insight into the genetic diversity of individual scabies infections. PLoS Negl Trop Dis.

[60] Pagel Van Zee J, Geraci NS, Guerrero FD, Wikel SK, Stuart JJ, Nene VM (2007). Tick genomics: the *Ixodes* genome project and beyond. Int J Parasitol.

[61] Jeyaprakash A, Hoy MA (2009). The nuclear genome of the phytoseiid *Metaseiulus occidentalis* (Acari: Phytoseiidae) is among the smallest known in arthropods. Exp Appl Acarol.

[62] Grbic M, Van Leeuwen T, Clark RM, Rombauts S, Rouze P, Grbic V (2011). The genome of *Tetranychus urticae* reveals herbivorous pest adaptations. Nature.

[63] Chan TF, Ji KM, Yim AK, Liu XY, Zhou JW, Li RQ (2015). The draft genome, transcriptome, and microbiome of *Dermatophagoides farinae* reveal a broad spectrum of dust mite allergens. J Allergy Clin Immunol.

[64] Morgan MS, Arlian LG, Rider SD, Grunwald WC, Cool DR (2016). A proteomic analysis of *Sarcoptes scabiei* (Acari: Sarcoptidae). J Med Entomol.

[65] Arlian LG, Morgan MS, Rider SD (2016). *Sarcoptes scabiei*: genomics to proteomics to biology. Parasit Vectors.

[66] Walton SF, Currie BJ, Kemp DJ (1997). A DNA fingerprinting system for the ectoparasite *Sarcoptes scabiei*. Mol Biochem Parasitol.

[67] Walton SF, Choy JL, Bonson A, Valle A, McBroom J, Taplin D (1999). Genetically distinct dog-derived and human-derived *Sarcoptes scabiei* in scabies-endemic communities in northern Australia. Am J Trop Med Hyg.

[68] Zhao Y, Cao Z, Cheng J, Hu L, Ma J, Yang Y (2015). Population identification of *Sarcoptes hominis* and *Sarcoptes canis* in China using DNA sequences. Parasitol Res.

[69] Zahler M, Essig A, Gothe R, Rinder H (1999). Molecular analyses suggest monospecificity of the genus *Sarcoptes* (Acari: Sarcoptidae). Int J Parasitol.

[70] Berrilli F, D'Amelio S, Rossi L (2002). Ribosomal and mitochondrial DNA sequence variation in *Sarcoptes* mites from different hosts and geographical regions. Parasitol Res.

[71] Gu XB, Yang GY (2008). A study on the genetic relationship of mites in the genus *Sarcoptes* (Acari: Sarcoptidae) in China. Int J Acarol.

[72] Alasaad S, Soglia D, Spalenza V, Maione S, Soriguer RC, Perez JM (2009). Is ITS-2 rDNA suitable marker for genetic characterization of *Sarcoptes* mites from different wild animals in different geographic areas?. Vet Parasitol.

[73] Amer S, El Wahab TA, Metwaly Ael N, Ye J, Roellig D, Feng Y (2014). Preliminary molecular characterizations of *Sarcoptes scabiei* (Acari: Sarcoptidae) from farm animals in Egypt. PLoS One.

[74] Walton SF, Dougall A, Pizzutto S, Holt D, Taplin D, Arlian LG (2004). Genetic epidemiology of *Sarcoptes scabiei* (Acari: Sarcoptidae) in northern Australia. Int J Parasitol.

[75] Andriantsoanirina V, Ariey F, Izri A, Bernigaud C, Fang F, Guillot J (2015). Wombats acquired scabies from humans and/or dogs from outside Australia. Parasitol Res.

[76] Andriantsoanirina V, Ariey F, Izri A, Bernigaud C, Fang F, Charrel R (2015). *Sarcoptes scabiei* mites in humans are distributed into three genetically distinct clades. Clin Microbiol Infect.

[77] Alasaad S, Soglia D, Sarasa M, Soriguer RC, Perez JM, Granados JE (2008). Skin-scale genetic structure of *Sarcoptes scabiei* populations from individual hosts: empirical evidence from Iberian ibex-derived mites. Parasitol Res.

[78] Alasaad S, Oleaga A, Casais R, Rossi L, Min AM, Soriguer RC (2011). Temporal stability in the genetic structure of *Sarcoptes scabiei* under the host-taxon law: empirical evidences from wildlife-derived *Sarcoptes* mite in Asturias, Spain. Parasit Vectors.

[79] Alasaad S, Fickel J, Rossi L, Sarasa M, Bena-Tez-Camacho B, Granados JE (2012). Applicability of major histocompatibility complex DRB1 alleles as markers to detect vertebrate hybridization: a case study from Iberian ibex x domestic goat in southern Spain. Acta Vet Scand.

[80] Rasero R, Rossi L, Soglia D, Maione S, Sacchi P, Rambozzi L (2010). Host taxon-derived *Sarcoptes* mite in European wild animals revealed by microsatellite markers. Biol Conserv.

[81] Alasaad S, Rossi L, Heukelbach J, Perez JM, Hamarsheh O, Otiende M (2013). The neglected navigating web of the incomprehensibly emerging and re-emerging *Sarcoptes* mite. Infect Genet Evol.

[82] Renteria-Solis Z, Min AM, Alasaad S, Muller K, Michler FU, Schmaschke R (2014). Genetic epidemiology and pathology of raccoon-derived *Sarcoptes* mites from urban areas of Germany. Med Vet Entomol.

[83] Oleaga A, Alasaad S, Rossi L, Casais R, Vicente J, Maione S (2013). Genetic epidemiology of *Sarcoptes scabiei* in the Iberian wolf in Asturias, Spain. Vet Parasitol.

[84] Gakuya F, Rossi L, Ombui J, Maingi N, Muchemi G, Ogara W (2011). The curse of the prey: *Sarcoptes* mite molecular analysis reveals potential prey-to-predator parasitic infestation in wild animals from Masai Mara, Kenya. Parasit Vectors.

[85] Gakuya F, Ombui J, Maingi N, Muchemi G, Ogara W, Soriguer RC (2012). Sarcoptic mange and cheetah conservation in Masai Mara (Kenya): epidemiological study in a wildlife/livestock system. Parasitology.

[86] Mellanby K (1972). Scabies.

[87] Arlian LG, Feldmeier H, Morgan MS (2015). The potential for a blood test for scabies. PLoS Negl Trop Dis.

[88] Arlian LG, Vyszenski-Moher DL, Gilmore AM (1988). Cross-antigenicity between *Sarcoptes scabiei* and the house dust mite, *Dermatophagoides farinae* (Acari: Sarcoptidae and Pyroglyphidae). J Med Entomol.

[89] Arlian LG, Vyszenski-Moher DL, Ahmed SG, Estes SA (1991). Cross-antigenicity between the scabies mite, *Sarcoptes scabiei*, and the house dust mite, *Dermatophagoides pteronyssinus*. J Invest Dermatol.

[90] Arlian LG, Rapp CM, Morgan MS (1995). Resistance and immune response in scabies-infested hosts immunized with *Dermatophagoides* mites. Am J Trop Med Hyg.

[91] Falk ES, Dale S, Bolle R, Haneberg B (1981). Antigens common to scabies and house dust mites. Allergy.

[92] Walton SF, Slender A, Pizutto S, Mounsey KE, Opresecu F, Thomas WR (2015). Analysis of IgE binding patterns to house dust mite allergens in scabies-endemic communities: insights for both diseases. Clin Exp Allergy.

[93] Arlian LG, Morgan MS, Estes SA, Walton SF, Kemp DJ, Currie BJ (2004). Circulating IgE in patients with ordinary and crusted scabies. J Med Entomol.

[94] Arlian LG, Morgan MS, Arends JJ (1996). Immunologic cross-reactivity among various strains of *Sarcoptes scabiei*. J Parasitol.

[95] Hollanders W, Vercruysse J, Raes S, Bornstein S (1997). Evaluation of an enzyme-linked immunosorbent assay (ELISA) for the serological diagnosis of sarcoptic mange in swine. Vet Parasitol.

[96] Kessler E, Matthes HF, Schein E, Wendt M (2003). Detection of antibodies in sera of weaned pigs after contact infection with *Sarcoptes scabiei* var. *suis* and after treatment with an antiparasitic agent by three different indirect ELISAs. Vet Parasitol.

[97] Lowenstein M, Kahlbacher H, Peschke R (2004). On the substantial variation in serological responses in pigs to *Sarcoptes scabiei* var. *suis* using different commercially available indirect enzyme-linked immunosorbent assays. Parasitol Res.

[98] van der Heijden HM, Rambags PG, Elbers AR, van Maanen C, Hunneman WA (2000). Validation of ELISAs for the detection of antibodies to *Sarcoptes scabiei* in pigs. Vet Parasitol.

[99] Rambozzi L, Menzano A, Lavin S, Rossi L. Biotin-avidin amplified ELISA for detection of antibodies to *Sarcoptes scabiei* in chamois (*Rupicapra *spp.). Vet Res. 2004;35(6):701–8. 10.1051/vetres:2004039 15535959

[100] Leon-Vizcaino L, Ruiz de Ybanez MR, Cubero MJ, Ortiz JM, Espinosa J, Perez L (1999). Sarcoptic mange in Spanish ibex from Spain. J Wildl Dis.

[101] Fernandez-Moran J, Gomez S, Ballesteros F, Quiros P, Benito JL, Feliu C (1997). Epizootiology of sarcoptic mange in a population of Cantabrian chamois (*Rupicapra pyrenaica parva*) in northwestern Spain. Vet Parasitol.

[102] Rodriguez-Cadenas F, Carbajal-Gonzalez MT, Fregeneda-Grandes JM, Aller-Gancedo JM, Huntley JF, Rojo-Vazquez FA (2010). Development and evaluation of an antibody ELISA for sarcoptic mange in sheep and a comparison with the skin-scraping method. Prev Vet Med.

[103] Bornstein S, Frossling J, Naslund K, Zakrisson G, Morner T (2006). Evaluation of a serological test (indirect ELISA) for the diagnosis of sarcoptic mange in red foxes (*Vulpes vulpes*). Vet Dermatol.

[104] Curtis CF (2001). Evaluation of a commercially available enzyme-linked immunosorbent assay for the diagnosis of canine sarcoptic mange. Vet Rec.

[105] Haas N, Wagemann B, Hermes B, Henz BM, Heile C, Schein E (2005). Crossreacting IgG antibodies against fox mite antigens in human scabies. Arch Dermatol Res.

[106] Oleaga A, Casais R, Gonzalez-Quiros P, Prieto M, Gortazar C (2008). Sarcoptic mange in red deer from Spain: improved surveillance or disease emergence?. Vet Parasitol.

[107] Casais R, Prieto M, Balseiro A, Solano P, Parra F, Martin Alonso JM (2007). Identification and heterologous expression of a *Sarcoptes scabiei* cDNA encoding a structural antigen with immunodiagnostic potential. Vet Res.

[108] Millan J, Casais R, Delibes-Mateos M, Calvete C, Rouco C, Castro F (2012). Widespread exposure to *Sarcoptes scabiei* in wild European rabbits (*Oryctolagus cuniculus*) in Spain. Vet Parasitol.

[109] He R, Shen N, Lin H, Gu X, Lai W, Peng X (2017). Molecular characterization of calmodulin from *Sarcoptes scabiei*. Parasitol Int.

[110] Mattsson JG, Ljunggren EL, Bergstrom K (2001). Paramyosin from the parasitic mite *Sarcoptes scabiei*: cDNA cloning and heterologous expression. Parasitology.

[111] Kuhn C, Lucius R, Matthes HF, Meusel G, Reich B, Kalinna BH (2008). Characterisation of recombinant immunoreactive antigens of the scab mite *Sarcoptes scabiei*. Vet Parasitol.

[112] Angelone-Alasaad S, Molinar Min A, Pasquetti M, Alagaili AN, D'Amelio S, Berrilli F (2015). Universal conventional and real-time PCR diagnosis tools for *Sarcoptes scabiei*. Parasit Vectors.

[113] Mounsey KE, McCarthy JS (2013). Treatment and control of scabies. Curr Opin Infect Dis.

[114] Liu X, Walton S, Mounsey K (2014). Vaccine against scabies: necessity and possibility. Parasitology.

[115] Thomas J, Peterson GM, Walton SF, Carson CF, Naunton M, Baby KE (2015). Scabies: an ancient global disease with a need for new therapies. BMC Infect Dis.

[116] Arlian LG, Morgan MS, Vyszenski-Moher DL, Stemmer BL (1994). *Sarcoptes scabiei*: the circulating antibody response and induced immunity to scabies. Exp Parasitol.

[117] Arlian LG, Morgan MS, Rapp CM, Vyszenski-Moher DL (1996). The development of protective immunity in canine scabies. Vet Parasitol.

[118] Tarigan S, Huntley JF (2005). Failure to protect goats following vaccination with soluble proteins of *Sarcoptes scabiei*: evidence for a role for IgE antibody in protection. Vet Parasitol.

[119] Arlian LG, Rapp CM, Vyszenski-Moher DL, Morgan MS (1994). *Sarcoptes scabiei*: histopathological changes associated with acquisition and expression of host immunity to scabies. Exp Parasitol.

[120] Morgan MS, Arlian LG (1994). Serum antibody profiles of *Sarcoptes scabiei* infested or immunized rabbits. Folia Parasitol (Praha).

[121] Harumal P, Morgan M, Walton SF, Holt DC, Rode J, Arlian LG (2003). Identification of a homologue of a house dust mite allergen in a cDNA library from *Sarcoptes scabiei* var. *hominis* and evaluation of its vaccine potential in a rabbit/*S. scabiei* Var. *canis* model. Am J Trop Med Hyg.

[122] Casais R, Granda V, Balseiro A, Del Cerro A, Dalton KP, Gonzalez R (2016). Vaccination of rabbits with immunodominant antigens from *Sarcoptes scabiei* induced high levels of humoral responses and pro-inflammatory cytokines but confers limited protection. Parasit Vectors.

[123] Uhlir J (1992). Immunization of rabbits with antigens from *Psoroptes cuniculi*, the rabbit scab mite. Folia Parasitol (Praha).

[124] Smith WD, Bates P, Pettit DM, Van Den Broek A, Taylor MA. Attempts to immunize sheep against the scab mite, *Psoroptes ovis*. Parasite Immunol. 2002;24(6):303–10. 10.1046/j.1365-3024.2002.00469.x 12102715

[125] Smith WD, Pettit DM (2004). Immunization against sheep scab: preliminary identification of fractions of *Psoroptes ovis* which confer protective effects. Parasite Immunol.

[126] Gu X, Xie Y, Wang S, Peng X, Lai S, Yang G (2014). Immune response induced by candidate *Sarcoptes scabiei* var. *cuniculi* DNA vaccine encoding paramyosin in mice. Exp Appl Acarol.

[127] Normaznah Y, Saniah K, Nazma M, Mak JW, Krishnasamy M, Hakim SL (1996). Seroprevalence of *Sarcoptes scabiei* var. *canis* antibodies among aborigines in peninsular Malaysia. Southeast Asian J Trop Med Public Health.

[128] Raez-Bravo A, Granados JE, Serrano E, Dellamaria D, Casais R, Rossi L (2016). Evaluation of three enzyme-linked immunosorbent assays for sarcoptic mange diagnosis and assessment in the Iberian ibex, *Capra pyrenaica*. Parasit Vectors.

[129] Rodriguez-Cadenas F, Carbajal-Gonzalez MT, Fregeneda-Grandes JM, Aller-Gancedo JM, Rojo-Vazquez FA (2010). Clinical evaluation and antibody responses in sheep after primary and secondary experimental challenges with the mange mite *Sarcoptes scabiei* var. *ovis*. Vet Immunol Immunopathol.

[130] Smets K, Vercruysse J (2000). Evaluation of different methods for the diagnosis of scabies in swine. Vet Parasitol.

[131] Vercruysse J, Geurden T, Peelaers I (2006). Development and Bayesian evaluation of an ELISA to detect specific antibodies to *Sarcoptes scabiei* var. *suis* in the meat juice of pigs. Vet Rec.

[132] Rampton M, Walton SF, Holt DC, Pasay C, Kelly A, Currie BJ (2013). Antibody responses to *Sarcoptes scabiei* apolipoprotein in a porcine model: relevance to immunodiagnosis of recent infection. PLoS One.

[133] Haas C, Rossi S, Meier R, Ryser-Degiorgis MP (2015). Evaluation of a commercial ELISA for the detection of antibodies to *Sarcoptes scabiei* in wild boar (*Sus scrofa*). J Wildl Dis.

[134] Davidson RK, Bornstein S, Handeland K (2008). Long-term study of *Sarcoptes scabiei* infection in Norwegian red foxes (*Vulpes vulpes*) indicating host/parasite adaptation. Vet Parasitol.

[135] Jakubek EB, Mattsson R, Morner T, Mattsson JG, Gavier-Widen D (2012). Potential application of serological tests on fluids from carcasses: detection of antibodies against *Toxoplasma gondii* and *Sarcoptes scabiei* in red foxes (*Vulpes vulpes*). Acta Vet Scand.

[136] Lower KS, Medleau LM, Hnilica K, Bigler B (2001). Evaluation of an enzyme-linked immunosorbent assay (ELISA) for the serological diagnosis of sarcoptic mange in dogs. Vet Dermatol.

[137] Bornstein S, Zakrisson G (1993). Humoral antibody response to experimental *Sarcoptes scabiei* var. *vulpes* infection in the dog. Vet Derm.

[138] Fuchs B, Zimmermann B, Wabakken P, Bornstein S, Mansson J, Evans AL (2016). Sarcoptic mange in the Scandinavian wolf *Canis lupus* population. BMC Vet Res.

[139] Sarasa M, Rambozzi L, Rossi L, Meneguz PG, Serrano E, Granados JE (2010). *Sarcoptes scabiei*: specific immune response to sarcoptic mange in the Iberian ibex *Capra pyrenaica* depends on previous exposure and sex. Exp Parasitol.

[140] Bornstein S, Zakrisson G (1993). Clinical picture and antibody response in pigs infected by *Sarcoptes scabiei* var. *suis*. Vet Derm.

[141] Zhang R, Jise Q, Zheng W, Ren Y, Nong X, Wu X (2012). Characterization and evaluation of a *Sarcoptes scabiei* allergen as a candidate vaccine. Parasit Vectors.

[142] Zhang R, Zheng W, Wu X, Jise Q, Ren Y, Nong X (2013). Characterisation and analysis of thioredoxin peroxidase as a potential antigen for the serodiagnosis of sarcoptic mange in rabbits by dot-ELISA. BMC Infect Dis.

[143] Zheng Y, He R, He M, Gu X, Wang T, Lai W (2016). Characterization of *Sarcoptes scabiei* cofilin gene and assessment of recombinant cofilin protein as an antigen in indirect-ELISA for diagnosis. BMC Infect Dis.

[144] Jayaraj R, Hales B, Viberg L, Pizzuto S, Holt D, Rolland JM (2011). A diagnostic test for scabies: IgE specificity for a recombinant allergen of *Sarcoptes scabiei*. Diagn Microbiol Infect Dis.

[145] Casais R, Goyena E, Martinez-Carrasco C, Ruiz de Ybanez R, Alonso de Vega F, Ramis G (2013). Variable performance of a human derived *Sarcoptes scabiei* recombinant antigen ELISA in swine mange diagnosis. Vet Parasitol.

[146] Casais R, Millan J, Rosell JM, Dalton KP, Prieto JM (2015). Evaluation of an ELISA using recombinant Ssλ20ΔB3 antigen for the serological diagnosis of *Sarcoptes scabiei* infestation in domestic and wild rabbits. Vet Parasitol.

[147] Oleaga A, Casais R, Balseiro A, Espi A, Llaneza L, Hartasanchez A (2011). New techniques for an old disease: sarcoptic mange in the Iberian wolf. Vet Parasitol.

[148] Dougall A, Holt DC, Fischer K, Currie BJ, Kemp DJ, Walton SF (2005). Identification and characterization of *Sarcoptes scabiei* and *Dermatophagoides pteronyssinus* glutathione S-transferases: implication as a potential major allergen in crusted scabies. Am J Trop Med Hyg.

